# The bioactive compounds, beneficial medicinal properties, and biotechnological prospects of *Fomitopsis*: a comprehensive overview

**DOI:** 10.3389/fcimb.2025.1534617

**Published:** 2025-04-22

**Authors:** Samantha C. Karunarathna, Nimesha M. Patabendige, Jaturong Kumla, Kalani K. Hapuarachchi, Nakarin Suwannarach

**Affiliations:** ^1^ Center for Yunnan Plateau Biological Resources Protection and Utilization, College of Biological Resource and Food Engineering, Qujing Normal University, Qujing, Yunnan, China; ^2^ School of Medical, Molecular and Forensic Sciences, Murdoch University, Perth, WA, Australia; ^3^ Center of Excellence in Microbial Diversity and Sustainable Utilization, Chiang Mai University, Chiang Mai, Thailand; ^4^ Department of Biology, Faculty of Science, Chiang Mai University, Chiang Mai, Thailand; ^5^ College of Biodiversity Conservation, Southwest Forestry University, Kunming, China

**Keywords:** bioactive compounds, traditional and modern medicine, therapeutic properties, enzymatic potential, biotechnological applications

## Abstract

Members of the genus *Fomitopsis* are medicinal mushrooms and a rich source of bioactive compounds with significant pharmacological and biotechnological potential. This paper provides a comprehensive review of their secondary metabolites, including polysaccharides, terpenoids, and phenolic compounds. In addition, their chemical structures and biological activities are described in detail. These compounds exhibit antioxidant, antimicrobial, anti-inflammatory, and immunomodulatory properties, with promising applications in cancer therapy, cardiovascular health, and immune modulation. Beyond medicine, *Fomitopsis* plays a crucial role in biotechnology, contributing to bioremediation, biofuel production, pharmaceutical development, and functional food innovation. By integrating traditional medicinal knowledge with recent scientific advances, this review highlights the biomedical significance and industrial relevance of *Fomitopsis*, underscoring its expanding role in health and environmental sustainability.

## 1 Introduction

**Figure 1:**
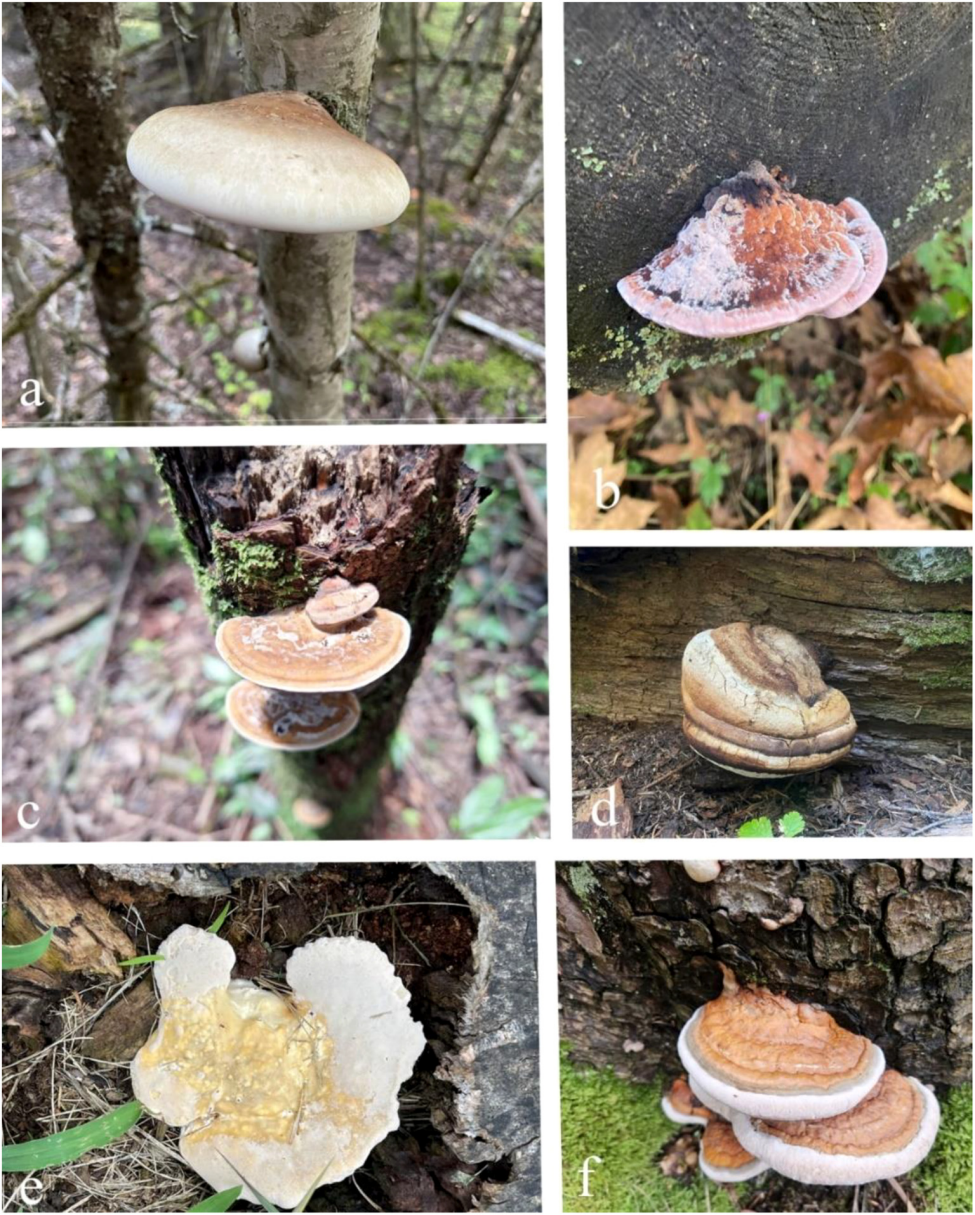
Some species of *Fomitopsis.* **(a)** *Fomitopsis betulina* **(b)** *F. cajanderi* **(c)** *F. feei* **(d)** *F. officinalis* **(e)** *F. Palustaris* **(f)** *F. Pinicola* (https://www.inaturalist.org/, the images are used under the license Attribution Non-Commercial-No Derivs 4.0).

The genus *Fomitopsis* comprises highly diverse fungi and encompasses species with unique ecological distributions and bioactive properties (Liu S. et al., 2022; Flores et al., 2023; Spirin et al., 2024; Gáper et al., 2025). Species such as *Fomitopsis betulina*, *F. cajanderi*, *F. feei*, *F. officinalis*, *F. palustris*, and *F. pinicola* (**Figure 1**) have been extensively studied for their medicinal and industrial potential (Li et al., 2024; Shen et al., 2024; Nowotarska et al., 2024). Initially recognized for their role in wood decomposition, these fungi are now celebrated as a rich source of bioactive compounds with significant therapeutic applications (Blanchette et al., 1992; Grienke et al., 2014; Girometta, 2019; Blanchette et al., 2021; Turner and Cuerrier, 2022; Hobbs, 2023; Flores et al., 2025). Advances in analytical techniques have revealed a wide range of pharmacologically active compounds, including polysaccharides, terpenoids, phenolic compounds, and secondary metabolites, all of which contribute to their ecological functions and therapeutic potential (Hsiao et al., 2003; Choi et al., 2007; Pleszczyńska et al., 2017; Zhao et al., 2018; Sułkowska-Ziaja et al., 2018; Tai et al., 2019; Muszyńska et al., 2020; Bishop, 2020; Sofrenić et al., 2021; Verekar et al., 2021; Gafforov et al., 2023; Zhang et al., 2023; Krupodorova et al., 2024).

Traditionally valued by indigenous cultures, species of *Fomitopsis* have been used for treating headache, nausea, and liver problems, as well as serving as haemostatics and anti-inflammatory agents due to their astringent effects. They were also employed for anti-fatigue, immune enhancement, cancer treatment, and as a styptic, antiseptic, and pain reliever across various regions., owing to the diverse bioactive compounds they produce (Hobbs, 1995; Grienke et al., 2014). These compounds exhibit potent antioxidant, antimicrobial, anti-inflammatory, and immunomodulatory effects, making them promising candidates for treating various medical conditions (Shamtsyan et al., 2004; Sulkowska-Ziaja et al., 2012; Li et al., 2013; Grienke et al., 2014; Peng et al., 2019; Fijałkowska et al., 2020; Kuo et al., 2021). In addition, *Fomitopsis* has shown significant potential in addressing chronic diseases such as cancer, cardiovascular disorders, and neurodegenerative conditions (Yoshikawa et al., 2005; Alresly et al., 2016, 2019; Bishop, 2020; Muszyńska et al., 2020; Badalyan et al., 2023). Members of this genus also offer innovative solutions to combat antibiotic-resistant pathogens, a pressing global health challenge (Girometta, 2019; Kumar and Prasher, 2022; Hashem et al., 2023). In addition, several *Fomitopsis*-derived products, including dietary supplements, functional foods, and extracts, are increasingly being developed and marketed for their purported health benefits, particularly in immune support and metabolic health.

Beyond medicine, *Fomitopsis* holds immense biotechnological promise. Its applications span bioremediation, biofuel production, pharmaceutical development, and functional food innovation (Tsujiyama and Okada, 2013; Pleszczyńska et al., 2017; Purnomo et al., 2020, 2022; Rehman et al., 2020; Mahmood et al., 2023; Prajapati et al., 2023). Some of the enzymes produced by *Fomitopsis* are particularly effective in breaking down lignocellulosic materials, offering sustainable solutions for biofuel production and environmental remediation (Kim H. et al., 2010; Park et al., 2015; Stipniece-Jekimova et al., 2022; Tiwari et al., 2023). In addition, their bioactive compounds are increasingly recognized as natural additives in the food industry, enhancing product quality and providing health benefits (Bishop, 2020; Grosse et al., 2020; Kozarski et al., 2022; Goppa et al., 2023). The multifaceted potential of *Fomitopsis*, including its bioactive compounds, therapeutic applications, and expanding role in biotechnology, are highlighted in this review.

## 2 Taxonomy and evolution of *Fomitopsis* species

The Fomitopsidaceae Jülich is one of the largest families of polypores, with twenty-four accepted genera (Liu et al., 2022, 2023). Most species are placed in the genus *Fomitopsis*, with 233 taxa being recognized (http://www.indexfungorum.Org, accessed 06 February 2025). However, recent studies based on multigene phylogenetic analysis have led to the acceptance of three genera (*Anthoporia*, *Antrodia*, and *Fomitopsis*) within the family Fomitopsidaceae. *Fomitopsis* now encompasses 128 species, including those formerly placed in the genera *Antrodia*, *Daedalea*, and *Laccocephalum* (Han et al., 2015; Spirin et al., 2024). *Fomitopsis* P. Karst. was originally introduced by Karsten, 1881, with *F. pinicola* (Sw.) P. Karst. designated as the type species (Kirk et al., 2008). *Fomitopsis* is widely distributed worldwide, highly polyphyletic, and serves as the type genus within the Fomitopsidaceae (Ortiz-Santana et al., 2013; Han et al., 2016; Liu et al., 2019, 2021). The species within the genus *Fomitopsis* are associated with brown rot, a process of significant ecological importance involving the decomposition and alteration of wood in forest ecosystems (Wei and Dai, 2004; Soares et al., 2017; Shah et al., 2018; Liu et al., 2023). Species of *Fomitopsis* produce basidiomata that range from sessile to effused-reflexed, growth patterns that range from annual to perennial, a spectrum of colors from white to purple, and a hyphal system that can be di- to trimitic, and features clamped generative hyphae. In addition, the basidiospores of species of *Fomitopsis* are characterized as smooth, hyaline, thin-walled, and may be subglobose to cylindrical in shape (Gilbertson and Ryvarden, 1986; Ryvarden and Gilbertson, 1993; Dai, 2012a; Li et al., 2013; Han and Cui, 2015; Haight et al., 2019).

## 3 Pathogenicity and ecological impact of *Fomitopsis* in forest ecosystems

*Fomitopsis* includes several species such as *F. nivosa* (Berk.) Gilb. & Ryvarden and *F. pinicola* Sw.) P. Kars that are known for their pathogenicity in forest ecosystems (Dai, 2012b; Liu S. et al., 2022). These fungi are primarily saprotrophic but can act as pathogens, causing significant decay in timber and living trees, affecting forest productivity and ecosystem health (Gramss, 2020; Zmitrovich et al., 2023; Pawłowicz et al., 2024). Species of *Fomitopsis* predominantly target conifers, with *F. pinicola* (commonly known as the red belt fungus) being notorious for causing brown rot in a variety of forest trees such as spruce, fir, and pine (Glaeser and Smith, 2016; Spirin et al., 2024). The pathogenicity of this fungus arises from its ability to decompose lignin selectively, leaving behind cellulose-rich residues. This leads to severe structural weakening of trees, making them susceptible to wind breakage and other environmental stressor factors (Singh and Singh, 2016; Haq et al., 2022; Waszczuk et al., 2022). Moreover, the persistence of *F. pinicola* in decayed wood can reduce timber quality, causing economic losses in forest industries (Hu, 2022; Sun et al., 2024).

Infections by *Fomitopsis* are facilitated by wounds on host trees, which serve as entry points for fungal spores. Once inside, the fungus colonizes the heartwood, initiating decay through enzymatic degradation of wood components (Schwarze et al., 1999; Adarsh et al., 2015; Pleszczyńska et al., 2017; Hu, 2022). The species of *Fomitopsis* involved produce a variety of enzymes, including cellulases, hemicellulases, and lignin-modifying enzymes, that help them break down the complex structure of wood, contributing to rapid degradation (Shah et al., 2018; Civzele et al., 2023). *Fomitopsis pinicola* also can colonize standing dead trees, stumps, and fallen logs, making it a key player in forest decomposition dynamics (Adarsh et al., 2015; Kauserud et al., 2024). Species of *Fomitopsis*, while pathogenic, also play essential roles in nutrient cycling within forest ecosystems. By breaking down woody material, they help release nutrients into the soil, aiding in forest regeneration (Rayner & Boddy, 1988; Pawłowicz et al., 2024; Ngwogu and Ngwogu, 2025).

However, the pathogenicity of species of *Fomitopsis* often outweighs their ecological benefits, especially in managed forests where timber quality and tree health are priorities (Schwarze and Baum, 2000; Gramss, 2020, Liu S. et al., 2022). In some cases, species of *Fomitopsis* can cause large-scale tree mortality, as observed in forests of the Pacific Northwest, where *F. pinicola* has been implicated in the widespread destruction and decline of coniferous forests (Hennon et al., 2002; U.S. Forest Service, 2023a, 2023b). Managing *Fomitopsis* infections in forests requires an integrated approach, including silvicultural practices that reduce tree stress and wound management to prevent fungal entry (Schwarze and Baum, 2000; Roberts et al., 2020; Dahlsjö, 2023). Chemical treatments including fungicides are often used in forestry to protect timber from fungal decay. However, biological control methods, including antagonistic fungi or bacteria, have shown promise in reducing the spread of *Fomitopsis* in forest ecosystems (Lonsdale et al., 2008; Hu, 2022, Griffin, 2024).

## 4 Major bioactive compounds in *Fomitopsis* and their beneficial medicinal properties

**Figure 2:**
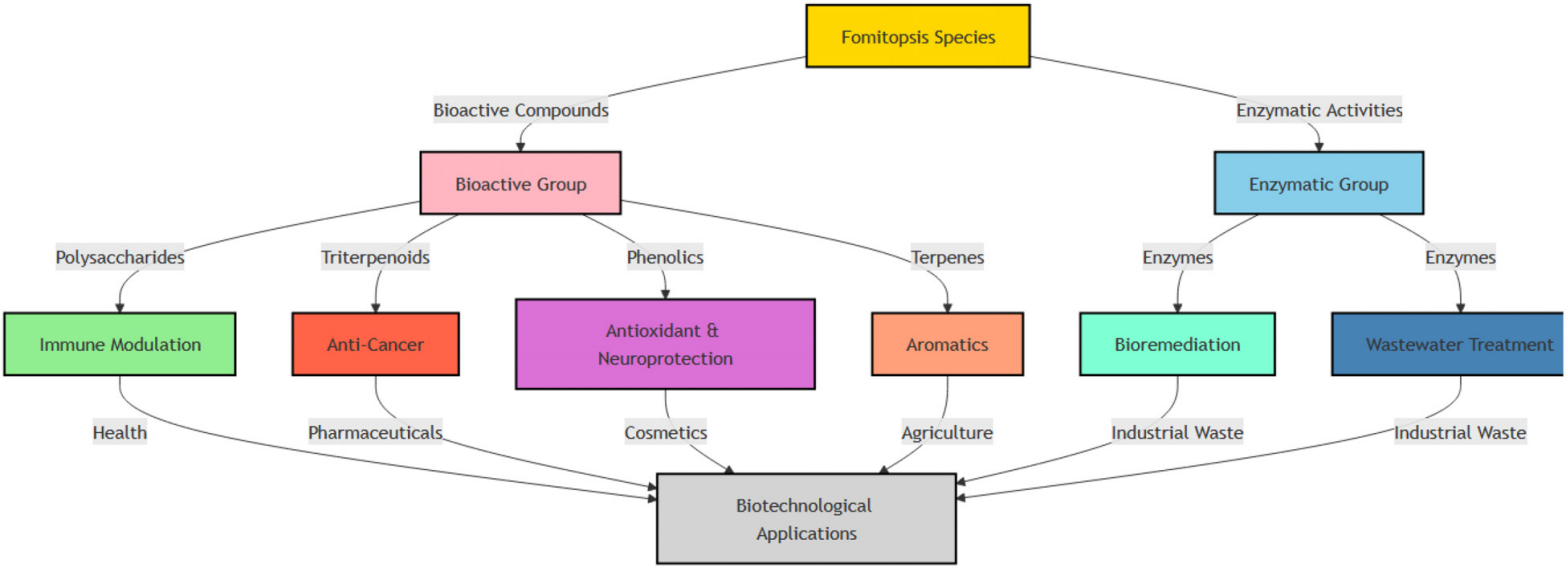
Bioactive compounds in species of *Fomitopsis* and their biotechnological applications in healthcare, cosmetics, agriculture, and industry.

Species of *Fomitopsis* produce a diverse array of bioactive compounds with significant biotechnological potential. These compounds include polysaccharides, triterpenoids, and phenolics. They play key roles in immune modulation, anticancer activity, antioxidant protection, neuroprotection, and aromatic applications. In addition, enzymatic activities contribute to such things as bioremediation and wastewater treatment. Together, these bioactivities support applications that involve pharmaceuticals, cosmetics, agriculture, and industrial waste management (**Figure 2**). The following section thoroughly explores these bioactive compounds, highlighting their specific properties and biotechnological applications.

### 4.1 Polysaccharides

#### 4.1.1 Fomitopsis betulina

The cellular structure of *F. betulina* contains beneficial polysaccharides, notably α-glucans, which exhibit water insolubility but can be dissolved in alkaline solutions (Grün, 2003; Pleszczyńska et al., 2017). Wiater et al. (2011) isolated and characterized (1→3)-α-d-glucans, with the main chain comprising 84.6% (1→3)-linked α-d-glucopyranose units along with 6% (1→4)-linked units. Piptoporane I was extracted and purified by Olennikov et al. (2012). The α-glucan in *F. betulina* primarily consists of (1→3)-α-d-glucopyranose units, with occasional branching by β-d-glucopyranose at the C6 position (17.3%). This specific type of fungal α-glucan, present in *F. betulina*, is known to stimulate the production of microbial mutanases, which have the potential to prevent dental caries. *Streptococci mutans* produces (1→3), (1→6)-α-d-Glucans (mutans), crucial components of dental plaque matrix, making them a promising target for enzymatic anti-caries strategies (Pleszczyńska et al., 2015). Nonetheless, *Streptococcal glucans* pose challenges as inducers of mutanases due to their low yield and structural variability. Birch polypore α-glucan, which can constitute up to 44–53% of the dry weight of the cell wall of *F. betulina* (Grün, 2003), offers a potential alternative to replace *S. glucans* (Wiater et al., 2008). α-(1 → 3)-Glucooligosaccharides (α-(1 → 3)-GOS), sourced from *F. betulina*, underwent evaluation for their ability to combat cancer. They demonstrated the capacity to hinder the growth of colon cancer cells by reducing their proliferation and promoting apoptosis, all the while leaving normal colon cells unaffected. These results suggest that α-(1 → 3)-GOS has potential as a valuable dietary or therapeutic substance for restraining cancer cell proliferation, especially in colon carcinoma models (Czerwonka et al., 2019). *Fomitopsis betulina* was assessed for mycelial growth and exopolysaccharide production across 22 strains. The study found significant variability in growth rates (3.50 ± 0.33 to 8.75 ± 0.50 mm/day) and exopolysaccharide production (0.02 ± 0.00 to 2.20 ± 0.31 g/L), with maltose as the optimal carbon source for growth and dextrose and starch enhancing exopolysaccharide yield. Notably, strain *F. betulina* 311 demonstrated strong growth and high biopolymer production, highlighting its biotechnological potential (Kizitska et al., 2024).

#### 4.1.2 Fomitopsis castaneus

The fermentation characteristics of exopolysaccharides (EPS) isolated from *F. castaneus* were studied in simulated human intestinal environments. The purified EPS, containing glucose, galactose, rhamnose, mannose, and arabinose, increased the production of short-chain fatty acids (SCFAs) in fecal extracts from both adults and children, with higher SCFA yields in children. Adding to the microbial flora, such as with *Enterococcus fecalis* and *Lactobacillus rhamnosus*, further enhanced SCFA production, highlighting the potential of EPS from *F. castaneus* to support gut health (Guo and Chi, 2017). FEPS, extracted from *Fomitopsis castanea* mycelia using ethanol precipitation, exhibited potent inhibition of mushroom tyrosinase with an IC50 of 16.5 mg/ml. It effectively reduced melanin production in human melanoma cells, diminished pigment density in embryos, and hindered NO production in macrophage cells with an IC50 of 42.8 ± 0.64 μg/ml (Jin et al., 2019).

#### 4.1.3 Fomitopsis cytisina

Three types of polygalacturonases, including two endo-type (EndoPG I, II) and one exo-type (ExoPG), were purified from *F. cytisina*. EndoPG I and II had molecular weights of 38 kDa, while ExoPG ranged from 50-60 kDa, with optimal pH values around 5.0-5.5 and thermal stability up to 45°C. EndoPGs showed varying activity on oligo-galacturonic acids, while ExoPG displayed maximum activity on substrates with 9 GalUA and no activity on unsaturated oligo-galacturonic acids (Miyairi et al., 2001).

#### 4.1.4 Fomitopsis officinalis

Mannofucogalactan, a major polysaccharide from *F. officinalis* fruiting bodies, was extracted using boiling water and purified, revealing a branched structure with a backbone of partially 3-O-methylated 1,6-O-linked α-D-galactopyranosyl residues. These residues were substituted at O-2 by 3-O-α-D-mannopyranosyl-α-L-fucopyranosyl and β-D-galactopyranosyl units, with α-L-fucopyranosyl units forming part of the side chains (Golovchenko et al., 2018).

Branched β-glucans from *F. officinalis* showed cytotoxic activity (Golovchenko et al., 2020). *Fomitopsis officinalis* yielded a purified heteropolysaccharide, FOBP50–1, with a molecular weight of 2.21 × 10^4^ g/mol, composed of 3-O-methylfucose, fucose, mannose, glucose, and galactose in a ratio of 1:6.5:4.4:8.1:18.2. Its structure was elucidated using UV, FT-IR, GC–MS, and NMR analysis. FOBP50–1 demonstrated significant antitumor activity in zebrafish assays by interacting with TLR-4, PD-1, and VEGF, thereby activating immunity and inhibiting angiogenesis. These results highlight its potential as a tumor immunotherapy agent (Shen et al., 2024). A purified heteropolysaccharide, FOBP90-1, was isolated from *F. officinalis* to explore its anticancer potential. FOBP90-1, with a molecular weight of 2.87 × 10^4^ g/mol, comprises several sugar residues, including α-d-Galp, α-l-Fucp, β-d-Glcp, α-d-Manp, and 3-O-Me-α-l-Fucp, as identified by UV, FT-IR, methylation analysis, and NMR. In zebrafish models, FOBP90-1 demonstrated anticancer activity by promoting immune activation and inhibiting angiogenesis. Mechanistic studies revealed that these effects were mediated through interactions with TLR-2, TLR-4, PD-L1, and VEGFR-2, suggesting FOBP90-1’s potential as a cancer treatment agent (Liu et al., 2024).

#### 4.1.5 Fomitopsis palustris

An extracellular β-glucosidase was purified from the brown-rot *F. palustris*, with a molecular mass of approximately 138 kDa and high homology with fungal β-glucosidases from glycosyl hydrolase family 3. The enzyme exhibited optimal activity at pH 4.5 and 70°C, with significant activity against p-nitrophenyl-β-d-glucoside and cellobiose, while being competitively inhibited by glucose and gluconolactone. These findings classify the β-glucosidase as an aryl-β-glucosidase with cellobiase activity, demonstrating notable thermostability (Yoon et al., 2008a).

#### 4.1.6 Fomitopsis pinicola

*Fomitopsis pinicola* is a traditional medicinal mushroom used in folk medicine in both China and Korea. Polysaccharides are the principal constituents of the fruiting body of *F. pinicola*. The extract (polysaccharide [FPP]) derived from *F. pinicola* was observed to lower fasting blood glucose levels while promoting increased body weight. In addition, FPP displayed a restorative impact on insulin levels in the bloodstream. Moreover, FPP demonstrated a noteworthy influence on lipid metabolism by reducing total cholesterol, triacylglycerol, and low-density lipoprotein cholesterol levels while elevating high-density lipoprotein cholesterol levels. Consequently, the FPP extract demonstrated beneficial properties for diabetes management, antioxidant effects, and regulation of lipid levels (Zahid et al., 2020a, 2020b, 2020c).

A heterogalactan was isolated from *F. pinicola* fruiting bodies and further separated into fucogalactan and mannofucogalactans using chromatography. Structural analysis revealed that all fractions are highly branched polysaccharides with a (1→6)-linked α-D-galactopyranosyl backbone, substituted with L-fucopyranosyl or mannopyranosyl-fucopyranose units (Usui et al., 1981). Polysaccharides from *F. pinicola* showed no toxicity to endothelial cells and had strong anti-angiogenic and anti-inflammatory effects (Cheng et al., 2008). *Fomitopsis pinicola* extract effectively lowered blood glucose levels by 77% after 20 days increasing HDL cholesterol by 73% and decreasing LDL cholesterol by 76%. This suggests its potential for atherosclerosis prevention and treatment, though more research is needed to understand the mechanisms involved (Cha et al., 2009). Researchers isolated *F. pinicola*, a potent ß-1, 4-glucosidase (BGL) producer through morphological and genetic analysis. They purified the BGL using a chromatographic process and found it belongs to glycoside hydrolase family 3, known for efficient enzymes. *F. pinicola* BGL stands out for its remarkable efficiency and specific substrate preferences (Joo et al., 2009). *Fomitopsis pinicola* polysaccharides comprise both extracellular and intracellular variants with distinct molecular weights, and both types of polysaccharides exhibited antioxidant properties, as evidenced by their ability to counteract DPPH and hydroxyl radicals *in vitro* and protect yeast cells from UV and hydrogen peroxide-induced oxidative damage. Importantly, intracellular polysaccharides displayed superior antioxidant activity compared to their extracellular counterparts (Hao et al., 2016).

*Fomitopsis pinicola* yielded twelve new sesquiterpenoids (fomitopins A–L [1–12]), through bioassay-guided purification. Their structures were determined using spectroscopic analyses and confirmed by ECD simulations. Ten compounds were tested for anti-inflammatory activity, with compound 11 showing the strongest inhibition of superoxide anion generation and elastase release (IC50 values of 0.81 ± 0.15 and 0.74 ± 0.12 μM). These sesquiterpenoids are promising candidates for further anti-inflammatory research (Tai et al., 2019). *Fomitopsis pinicola* exhibits protective effects against alcohol-induced liver injury through its mycelia polysaccharides (FPMPS). FPMPS improved serum lipid levels, maintained hepatic and cecal morphology, and modulated gut microbiota disrupted by alcohol. Mechanistically, FPMPS-regulated pathways, such as retinol metabolism, bile secretion, TRP channel inflammation, and the PI3K-Akt signaling pathway, play a key role in preventing liver damage. FPMPS shows promise as a potential therapeutic agent for acute alcoholic liver injury and as a functional health food (Wu et al., 2023). **Table 1** lists polysaccharides from various species of *Fomitopsis*, along with their structures and therapeutic applications.

**Table 1:** Polysaccharides, structures, and therapeutic applications of various species of *Fomitopsis*.

Species	Polysaccharide	Structure/Characteristics	Effects/Applications	Reference
*Fomitopsis betulina*	α-(1 → 3)-Glucooligosaccharides (α-(1 → 3)-GOS)	α-(1 → 3)-linked α-d-glucopyranose units	Inhibits colon cancer cell growth, promotes apoptosis	Czerwonka et al. (2019)
	α-Glucans	Water-insoluble, dissolved in alkaline solutions	Promotes microbial mutanases for preventing dental caries	Pleszczyńska et al. (2017)
	(1→3)-α-d-Glucans	α-(1→3)-linked glucopyranose with β-d-glucopyranose branching	Stimulates mutanase production for dental plaque prevention	Wiater et al. (2011)
	Exopolysaccharides	Significant variability in growth and exopolysaccharide production	Biotechnological potential (notably strain F. betulina 311)	Kizitska et al. (2024)
*F. castane*	Exopolysaccharides (FEPS)	Strong mushroom tyrosinase inhibition (IC50 16.5 mg/mL)	Reduces melanin production in melanoma cells, anti-inflammatory effects	Jin et al. (2019)
	Exopolysaccharides (EPS)	Contains glucose, galactose, rhamnose, mannose, arabinose	Promotes SCFA production in human intestinal environments	Guo and Chi (2017)
*F. cytisina*	Polygalacturonase (EndoPG I, II, ExoPG)	EndoPG I, II: 38 kDa, ExoPG: 50-60 kDa	Optimal activity at pH 5.0-5.5, varying activity on oligo-galacturonic acid	Miyairi et al. (2001)
*F. officinalis*	Mannofucogalactan	Branched α-D-galactopyranosyl structure	Cytotoxic activity	Golovchenko et al. (2020)
	FOBP50–1 (Heteropolysaccharide)	3-O-methylfucose, fucose, mannose, glucose, galactose	Antitumor activity, immune activation, angiogenesis inhibition	Shen et al. (2024)
	FOBP90-1 (Heteropolysaccharide)	α-d-Galp, α-l-Fucp, β-d-Glcp, α-d-Manp, 3-O-Me-α-l-Fucp	Anticancer activity, immune activation, angiogenesis inhibition	Liu et al. (2024)
*F. pinicola*	Heterogalactan (fucogalactan, mannofucogalactans)	(1→6)-linked α-D-galactopyranosyl backbone with L-fucopyranosyl or mannopyranosyl-fucopyranose units	Therapeutic potential	Usui et al. (1981)
	Polysaccharides	Lowered fasting blood glucose levels, improved lipid metabolism, antioxidant effects	Diabetes management, antioxidant activity	Zahid et al. (2020a, 2020b, 2020c)
	EMFP (Extract)	Contains phenolic compounds and triterpene	Antioxidant and anticancer effects, inhibited HepG2 proliferation	Zhang et al. (2023)
	FPMPS (Mycelia Polysaccharides)	Modulates gut microbiota, regulates lipid levels, TRP channel inflammation, PI3K-Akt signaling pathway	Protective against alcohol-induced liver injury	Wu et al. (2023)
	Sesquiterpenoids (fomitopins A–L)	Sesquiterpenoid structures	Anti-inflammatory effects (strong inhibition of superoxide anion generation, elastase release)	Tai et al. (2019)
	Polysaccharides	Strong antioxidant, anti-angiogenic, and anti-inflammatory effects	Potential therapeutic agents	Cheng et al. (2008)
	Polysaccharides (Intracellular and extracellular)	Distinct molecular weights	Antioxidant properties, protection against UV and oxidative damage	Hao et al. (2016)
	ß-1, 4-glucosidase (BGL)	Glycoside hydrolase family 3 enzyme	Efficient ß-glucosidase production	Joo et al. (2009)
*F. palustris*	β-Glucosidase	Molecular mass of ~138 kDa, fungal β-glucosidase (glycosyl hydrolase family 3)	Optimal activity at pH 4.5 and 70°C, cellobiase activity	Yoon et al. (2008a)

### 4.2 Terpenoids

**Figure 3:**
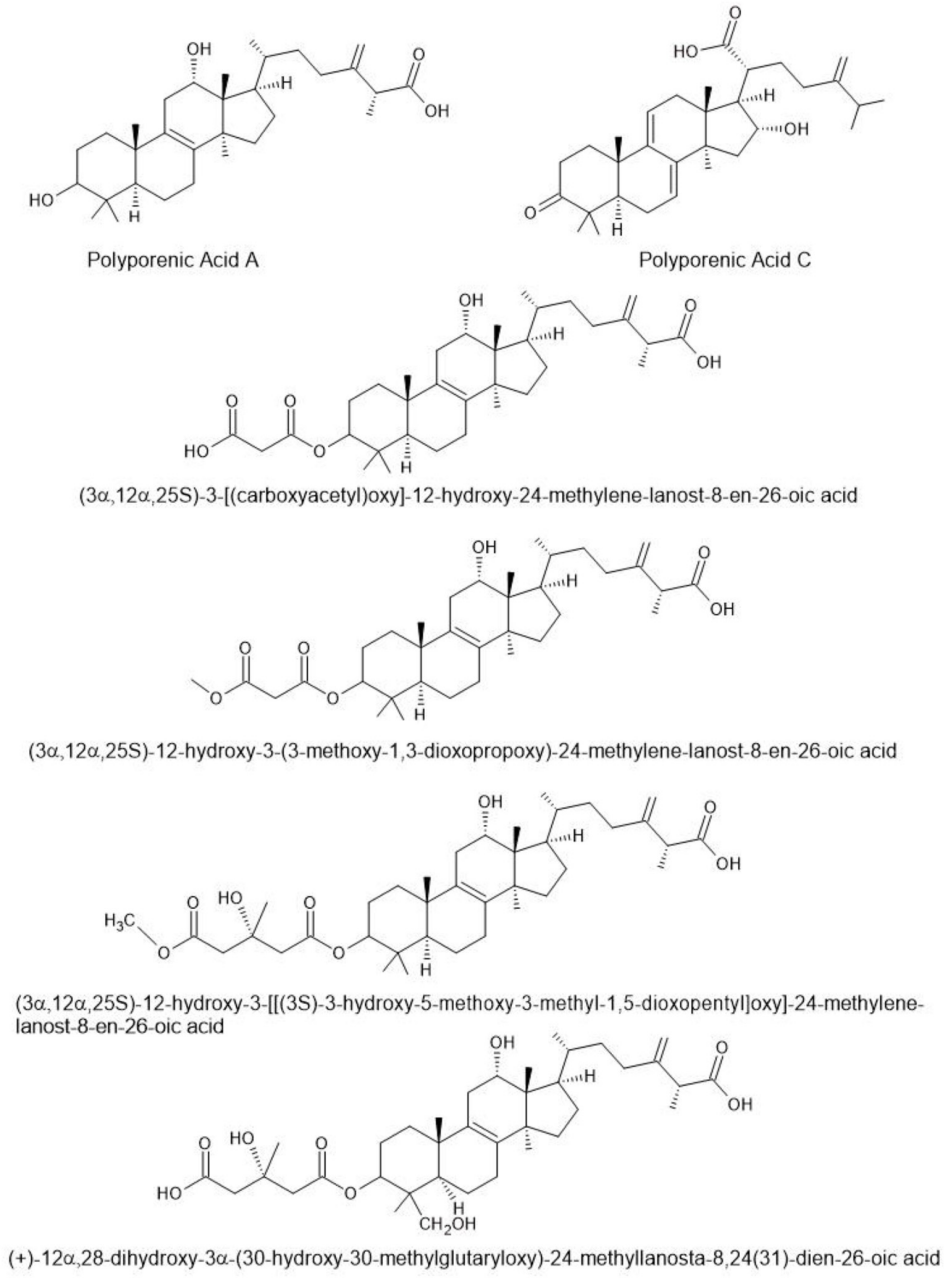
Triterpenoids found in species of *Fomitopsis* (Pleszczyńska et al., 2017).

Terpenoids are a large and diverse class of natural compounds known for their structural variety and biological activities. In species of *Fomitopsis*, these compounds (**Figure 3**) are particularly notable for their antimicrobial, anti-inflammatory, and therapeutic properties. In this section, we discuss recent research on *Fomitopsis* terpenoids, highlighting their chemical diversity and potential applications in medicine and biotechnology.

#### 4.2.1 Fomitopsis betulina

The mycelium of *F. betulina* was cultured via liquid fermentation, leading to the isolation of seven pimarane-type diterpenes from the fermentation broth. These were pipulinus A–D, pipulinus F, elaeicolasides B, and pipulinus E (Rapior et al., 1996). Twenty sesquiterpene compounds were identified from fresh *F. betulina* fruiting bodies, including (R)-trans-nerolidol, β-elemene, and α-chamigrene. Four of these—Isobazzanene, (S)-(−)-daucene, (−)-β-barbatene, and (+)-α-barbatene—were reported as fungal components for the first time. Subsequent research identified four additional monoterpenes such as linalool and α-terpineol, further expanding the understanding of *F. betulina*’s volatile constituents (Rösecke et al., 2000). Schlegel et al. (2000) found piptamine in *F. betulina*, which exhibited antimicrobial properties against bacteria and yeast, including *C. albicans*, at low concentrations. This discovery highlights the significance of compounds beyond polysaccharides in mushroom bioactivity. A novel phenolic compound, (E)-2-(4-hydroxy-3-methyl-2-butenyl)-hydroquinone, was isolated from the fresh fruiting bodies of *F. betulina* (Kawagishi et al., 2002).

Polyporenic acid C and three additional triterpenoids found in *F. betulina* have been shown to exhibit anti-inflammatory and antibacterial properties by proficiently blocking the activity of 3α hydroxysteroid dehydrogenase and bacterial hyaluronate lyase (Wangun et al., 2004). Two significant tetraterpene compounds, *β*-carotene, and lycopene, were successfully identified in the dried powder of *F. betulina* fruiting bodies. These compounds are recognized for their potent antioxidant properties. The known spectrum of phenolics in this species was expanded by detecting four tocopherols—α-tocopherol, β-tocopherol, γ-tocopherol, and δ-tocopherol—in a dried powder of *F. betulina* (Reis et al., 2011). Alresly et al. (2016) isolated one new and ten known triterpenes from fungal fruiting bodies of *F. betulina*. The new compound had antibacterial activity. *Fomitopsis betulina* showed high potential for antitumor activities (Sari et al., 2020). In a rich fermentation broth of *F. betulina*, three sesquiterpenes, including cryptosphaerolide B and two bicyclic sesquiterpenes, rel-(1S,4S,5R,7R,10R)-10-desmethyl-11-euduemene and 10,11-epoxyguaian-13-ol, were identified. In addition, seven pimarane-type diterpenes, pipulinus A–D, pipulinus F, elaeicolasides B, and pipulinus E, were isolated from the mycelium cultured via liquid fermentation. Two phenolic compounds, (3R)-5-carbomethoxymellein and 4-hydroxyphenethyl alcohol, were also identified in the fermentation broth (Sun, 2015). Five previously unreported lanostane-type triterpenoids, named piptolinic acids A–E, were identified from *F. betulina*, along with five known lanosterol-type triterpenoids: 3-epi-(3′-hydroxy-3′-methylglutaryloxyl)-dehydrotumulosic acid, dehydroeburiconic acid, 6α-hydroxypolyporenic acid C, and 3-epi-dehydropachymic acid (Tohtahon et al., 2017). Previously unreported 24-methyl-lanostane-type triterpenes (piptolinic acid F–J) were isolated from dried *F. betulina* fruiting bodies, along with seven known lanosterol-type triterpenoids (Khalilov et al., 2018). Eleven triterpenoids and betulin from *F. betulina* were tested for protective effects against chromosome aberrations in human lymphocytes using the CBMN assay. Most compounds reduced DNA damage more effectively than amifostine, with 2.0 µg/mL being the most effective concentration. D8-lanostanes exhibited better activity than those with a conjugated 7,9 (11)-diene system, while betulin showed the lowest protective activity, comparable to amifostine (Anđelković et al., 2021). Thirteen new 24-methylene lanostane triterpenoids and seventeen previously identified compounds were isolated from *F. betulina*. Fomitosides L and N exhibited cytotoxic effects on HL60 leukemia cells. Among the known compounds, dehydropachymic acid, pachymic acid, 3-epi-dehydrotumulosic acid, and 12α-hydroxy-3α-(3’-hydroxy-4’-methoxycarbonyl-3’-methylbutyryloxy)-24-methyllanosta-8,24(31)-dien-26-oic acid demonstrated significant cytotoxicity against HL60 leukemia cells while showing selectivity for MRC-5 healthy cells (Sofrenić et al., 2021). Researchers have identified 47 different lanostane-type triterpenoids in *F. betulina* (Li et al., 2024).

#### 4.2.2 Fomitopsis officinalis

*Fomitopsis officinalis* fruiting bodies contain abundant triterpenoids, polysaccharides, organic acids, coumarins, and phenolic compounds. Scientific studies have shown that extracts and isolated components from *F. officinalis* offer diverse therapeutic advantages, encompassing anti-inflammatory, cytotoxic, and antimicrobial properties (Muszyńska et al., 2020). Antiviral effects of *F. officinalis* could prevent neuropathies associated with infections caused by the herpes viruses or hepatitis C (Stamets, 2011). Two chlorinated coumarins were isolated from the ethanol extract of *F. officinalis* and characterized. These compounds demonstrated specific antimicrobial activity against *M. tuberculosis*, including drug-resistant isolates, with minimum inhibitory concentrations (MICs) ranging from 22 to 50 µg/ml (Hwang et al., 2012). Eight compounds were isolated from *F. officinalis*, including 4, 6, 8 (14), 22 (23)-tetraen-3-one-ergostane, identified for the first time. Fomefficinic acids A and C and 3-keto-dehydrosulfurenic acid inhibited MCF-7 breast cancer cells, while fomefficinic acids A and C also suppressed SMMC-7721 liver cancer cells (Chi et al., 2014). Triterpene lactone, known as fomefficin, and the sesquiterpene extracted from *F. officinalis* have demonstrated notable anti-cancer effects (Feng and Yang, 2015). Similar properties have been observed in a group of triterpene compounds called the officimalonic acids A – H isolated from the methanol extract of *F. officinalis*. Their anti-inflammatory and cytotoxic activity in *in vitro* conditions was confirmed in contact with human cancer cells H460, HepG2, and BGC–823 (Wu et al., 2014). The exploration of the chemical composition of *F. officinalis* resulted in the isolation of four newly identified lanostane triterpenoids and four previously reported triterpenoids. Their capacity to inhibit *Trypanosoma congolense*, a pathogenic agent causing severe animal diseases, was assessed. Compounds 2-5 and 8 demonstrated moderate inhibitory activity, with IC50 values ranging from 7.0-27.1 µM (Naranmandakh et al., 2018). Flavonoids in *F. officinalis* can potentially mitigate oxidative stress in the aging mouse brain (Sha, 2016). Dehydrosulfurenic acid, a lanostane-type triterpenoid derived from *F. officinalis*, has been patented for its potential use as a pharmaceutical treatment for ischemic stroke (Saba et al., 2015; Simi and Prisco, 2018; Flores et al., 2023),. Moreover, eburicoid acid could further prove to have antidepressant effects (Yang et al., 2019; Muszyńska et al., 2020).

#### 4.2.3 Fomitopsis pinicola

In this study, the fresh fruiting body of *F. pinicola* was freeze-dried, and 300g of the dried mushroom was extracted sequentially with dichloromethane and methanol. The dichloromethane extract, accounting for 13.7% of the total extract, exhibited antimicrobial activity in a TLC bioassay (Hamburger & Cordell, 1987). Phytochemical analysis of methanol and n-hexane extracts from *F. pinicola* identified triterpene derivatives and aromatic compounds reduced from lignin. New natural compounds such as pinicolol C and pinicolic acid E were discovered, along with steryl esters such as 3β-linoleyloxyergosta derivatives. HPLC and TLC confirmed that the surface of *F. pinicola* is rich in lanostane derivatives (Rösecke and König, 2000). Two newly discovered lanostane triterpenoids and ten previously unreported lanostane triterpene glycosides were found in *F. pinicola* (Yoshikawa et al., 2005). A novel lanostane triterpene, 3α-acetyloxylanosta-8,24-diene-21-ol, was obtained from an active fraction of the fungus *F. pinicola* extract. In addition, two known triterpenic acids, pinicolic acid A and 3α-acetoxylanosta-8, 24-dien-21-oic acid, were identified. These compounds demonstrate cytotoxic and antimicrobial activities (Petrova et al., 2007). Various lanostane triterpenoids and an ergostane compound obtained from American *F. pinicola* exhibited antimicrobial activity, particularly against *B. cereus*, with MIC values ranging from 16 to 128 μg/ml. Compounds A, B, C, and F demonstrated specific antimicrobial effects, while D and E displayed lower specificity (Liu et al., 2010). The effects of *F. pinicola*, a medicinal fungus with notable anti-tumor activity primarily due to 3α-acetoxylanosta-8,24-dien-21-oic acid, were assessed on immune function in mice. The study found increased phagocytic rate, serum hemolysin levels, lymphocyte conversion rate, and phagocytic index in the *F. pinicola* group compared to controls. The compound enhanced lymphocyte transformation at low concentrations, but the effect decreased at higher concentrations. Overall, dry mycelia of *F. pinicola* effectively enhanced immune competence in mice (Bao et al., 2015).

To identify the chemical constituents of the fruiting bodies of *F. pinicola*, researchers isolated a novel lanostane triterpene glycoside, named fomitoside K, from its methanolic extract (Lee et al., 2012). Three new 24-methyl-lanostane triterpenoids (fomitopsins D-F) and four known compounds were isolated from *F. feei*. Fomitopsins E and F showed antibacterial activity against *B. cereus*, while fomitopsin D exhibited antiviral activity against herpes simplex virus type 1 (HSV-1) (Isaka et al., 2017). Seven lanostane-type triterpenes isolated from *F. pinicola* and *F. officinalis* showed significant antitumor activity, particularly in MCF-7 cells. Compounds 2 and 4 effectively suppressed tumor growth in mice, influencing VEGF and cytokine expression. Acetyl or carbonyl at C-3 and hydroxy at C-15 enhanced their antitumor effects (Shi et al., 2017). A triterpenoid, 3 acetoxylanosta 8,24 dien 21 oic acid (FPOA), a triterpenoid obtained from the fruiting body of *F. pinicola*, exhibits cytotoxic properties, particularly targeting HepG2 hepatoma cells (Song et al., 2018). *Fomitopsis pinicola* yielded twelve new sesquiterpenoids, fomitopins A–L (1–12), through bioassay-guided purification. The structures were determined using spectroscopic analyses and confirmed by ECD simulations. Ten compounds were tested for anti-inflammatory activity, with compound 11 showing the strongest inhibition of superoxide anion generation and elastase release (IC50 values of 0.81 ± 0.15 and 0.74 ± 0.12 μM). These sesquiterpenoids are promising candidates for further anti-inflammatory research (Tai et al., 2019). A methanolic extract of *F. pinicola* yielded 35 lanostane-type triterpenoids, including 13 newly discovered compounds and 22 previously known ones. These compounds demonstrated cytotoxicity against various human tumor cell lines, such as HL-60, A549, SMMC-7721, MCF-7, and SW480. In addition, certain compounds exhibited selective inhibitory effects against specific cell lines, with some inducing apoptosis in HL-60 cells (Peng et al., 2019). Twelve previously unreported sesquiterpenoids, named fomitopins A–L (1–12), were isolated from *F. pinicola*, known for its antimicrobial and anti-inflammatory properties. These newly discovered sesquiterpenoids hold promise for further anti-inflammatory research (Tai et al., 2019). Fruiting bodies of *F. pinicola* yielded 28 lanostane triterpenoids, comprising 11 novel and 17 previously identified compounds. Some of these compounds reduced nitric oxide release, while others demonstrated notable PTP1B inhibitory properties. Kinetic analysis confirmed two compounds as competitive PTP1B inhibitors, and three were observed to enhance glucose uptake in insulin-resistant cells. These findings indicate the potential of *F. pinicola* as a functional food or medicine for diabetes management (Zhang et al., 2020).

Thirteen novel and nine known lanostane triterpenoids were isolated from *F. pinicola* fruiting bodies, with their structures confirmed through spectroscopic analysis and X-ray diffraction. Nor-pinicolic acids A−F, featuring unique C-25-C-27 nor-lanostane skeletons, were first identified in this species. Anti-inflammatory assays showed that pinicopsic acid F and 16α-hydroxy-3-oxolanosta-7,9(11),24-trien-21-oic acid exhibited moderate inhibition of LPS-induced NO production in RAW 264.7 cells, with IC50 values of 24.5 and 25.7 μM (Liu et al., 2022).

Twelve previously unreported lanostane-type triterpenes, along with twenty-two known triterpenes, were discovered and characterized in *F. pinicola*. Thirty-two triterpene compounds were assessed for their anti-inflammatory potential using neutrophils as a model, with pinicolasin J emerging as the most effective inhibitor of superoxide anion generation (Kuo et al., 2021). Volatile compounds from *F. pinicola* were analyzed, and during sporulation, *F. pinicola* released (R)- and (S)-oct-1-en-3-ol, octan-3-one, and sesquiterpenes. Chopping up the fruiting bodies released volatiles and they attracted wood-living beetles and moths, with rac-oct-1-en-3-ol being a key attractant (Fäldt et al., 1999). Phytochemical analysis of *F. pinicola* yielded a new lanostanoid derivative and seven known triterpenes. Five of these compounds showed antimicrobial activity against *Bacillus subtilis* (Keller et al., 1996).

#### 4.2.4 Other *Fomitopsis* species

Chemical analysis of *Fomitopsis carnea* solid-state cultures led to the discovery of two new triterpenoid glycosides, forpiniosides B and C, and two already known compounds. Structural identification was carried out using HRESIMS and NMR techniques. Among the compounds, forpinioside B showed notable antimicrobial activity against *Staphylococcus aureus* and *B. subtilis*, with MIC values comparable to standard antibiotics gentamycin and oxytetracycline (Sum et al., 2023). A new lanostane triterpene glycoside, fomitoside-K, was isolated from *Fomitopsis nigra* and tested for anticancer activity against human oral squamous cell carcinoma (YD-10B) cells. Fomitoside-K induced apoptosis via mitochondrial dysfunction, increased ROS levels, and activated JNK and ERK pathways. Its effects were reduced by ROS scavengers and MAPK inhibitors. In addition, fomitoside-K showed synergy with adriamycin, suggesting its potential as a treatment for oral cancer through a ROS-dependent mitochondrial apoptosis pathway (Bhattarai et al., 2012). Hypercholesterolemia, a major risk factor for coronary heart disease, can be mitigated by inhibiting NPC1L1-mediated cholesterol absorption. A novel compound, fomiroid A, was discovered in *F. nigra* mushroom extracts, showing potent inhibition of ezetimibe glucuronide binding to NPC1L1. Fomiroid A, a lanosterone derivative (C_30_H_48_O_3_), dose-dependently blocked cholesterol uptake in Caco2 cells and acted as a pharmacological chaperone for the L1072T/L1168I mutant of NPC1L1, suggesting a unique mechanism of action compared to ezetimibe (Chiba et al., 2014).

The extract from *Fomitopsis rosea* yielded two novel lanostane triterpenes—3α-(3′-butylcarboxyacetoxy) oxepanoquercinic acid C 1 and 3α-hydroxy-24-methylene-23-oxolanost-8-en-26-carboxylic acid 2—in addition to three previously identified triterpenes and one epidioxy sterol derivative. While all these triterpenes demonstrated antibacterial effects against *S. aureus*, none exhibited anti-radical properties against DPPH radicals (Popova et al., 2009). *Fomitopsis* sp*raguei* was investigated for its methanolic extract, leading to the isolation of five lanostane-type triterpenoids. These included three novel compounds named fomitopsins A–C, along with two known compounds: quercinic acid C and 3α-carboxyacetyl-12β-hydroxyquercinic acid (Quang et al., 2005). Two newly identified 24-methyl-lanostane triterpenoids, named fomitopsins I and J, were detected in *Fomitopsis* sp. along with seven previously recognized compounds. One known compound exhibited antibacterial effects against *B. cereus* (with a minimum inhibitory concentration of 6.25 μg/ml) and *Enterococcus faecium* (with a minimum inhibitory concentration of 12.5 μg/ml) (Isaka et al., 2019). The triterpenoid composition of various *Fomitopsis* species, highlighting their bioactivities, including anticancer, anti-inflammatory, and antimicrobial properties is summarized in **Table 2**.

**Table 2:** Summary of results from triterpenoid analysis and bioactivity findings related to various species of *Fomitopsis*.

Species	Compound/Type of Extract	Biological Activity/Effect	Reference
*Fomitopsis carnea*	Forpiniosides B and C	Antimicrobial activity against *S. aureus* and *B. subtilis* (MIC comparable to antibiotics)	Sum et al. (2023)
*F. betulina*	Fomitosides L and N	Cytotoxic effects on HL60 leukemia cells	Sofrenić et al. (2021)
	Dehydropachymic acid, pachymic acid, 3-epi-dehydrotumulosic acid, 12α-hydroxy-3α-(3’-hydroxy-4’-methoxycarbonyl-3’-methylbutyryloxy)-24-methyllanosta-8,24(31)-dien-26-oic acid	Significant cytotoxicity against HL60 leukemia cells; selectivity for MRC-5 healthy cells	Sofrenić et al. (2021)
	Polyporenic acid C	Anti-inflammatory and antibacterial by blocking activity of 3α hydroxysteroid dehydrogenase and bacterial hyaluronate lyase	Wangun et al. (2004)
	Pimarane-type diterpenes	Identified from liquid fermentation; includes pipulinus A–D, pipulinus F, elaeicolasides B, pipulinus E	Rapior et al. (1996)
	Volatile constituents	Identified 20 sesquiterpenes, including new fungal components and additional monoterpenes	Rösecke et al. (2000)
	Piptamine	Antimicrobial properties against bacteria and yeast, including *Candida albicans*	Schlegel et al. (2000)
	β-carotene and lycopene	Potent antioxidant properties	Reis et al. (2011)
	Tocopherols	Expanded known spectrum of phenolics	Reis et al. (2011)
	Cryptosphaerolide B	Identified in rich fermentation broth; sesquiterpene compounds	Sun (2015)
	Piptolinic acids A–E	Five previously unreported lanostane-type triterpenoids	Tohtahon et al. (2017)
	Various triterpenoids	Protective effects against chromosome aberrations; most compounds reduced DNA damage effectively	Anđelković et al. (2021)
	Piptolinic acid F–J	Newly isolated; combined with known triterpenoids	Khalilov et al. (2018)
*F. feei*	Fomitopsins D-F	Antibacterial activity against *B. cereus*, antiviral activity against HSV-1	Isaka et al. (2017)
*F. officinalis*	Compounds from *F. officinalis*	Diverse therapeutic advantages; anti-inflammatory, cytotoxic, antimicrobial properties	Muszyńska et al. (2020)
	Triterpene lactone (fomefficin)	Notable anti-cancer effects	Feng and Yang (2015)
	Officimalonic acids A–H	Anti-inflammatory and cytotoxic activity against human cancer cells	Wu et al. (2014)
	Dehydrosulfurenic acid	Potential use for ischemic stroke treatment	Saba et al. (2015); Simi and Prisco (2018); Flores et al. (2023)
*F. palustris*	New compounds from *F. palustris*	Polyporenic acid B showed strong cytotoxicity against cancer cell lines	Zhao et al. (2018)
*F. pinicola*	Nor-pinicolic acids A–F	Moderate inhibition of LPS-induced NO production in RAW 264.7 cells	Liu Y. et al. (2022)
	Fomitopins A–L (1–12)	Anti-inflammatory activity; compound 11 shows strongest inhibition of superoxide anion generation and elastase release	Tai et al. (2019)
	Lanostane-type triterpenoids	Cytotoxicity against various human tumor cell lines	Peng et al. (2019)
	3-acetoxylanosta-8,24-dien-21-oic acid (FPOA)	Cytotoxic properties targeting HepG2 hepatoma cells	Song et al. (2018)
	Piptolinic acid F–J	Newly isolated lanostane triterpene glycoside	Lee et al. (2012)
	Nitric oxide release inhibitors	Compounds exhibit notable PTP1B inhibitory properties; potential for diabetes management	Zhang et al. (2020)
	Antimicrobial activity	Compounds show specific antimicrobial effects against *B. cereus*	Liu et al. (2010)

### 4.3 Proteins/Enzymes

#### 4.3.1 Fomitopsis meliae

A novel thermostable endoglucanase has been identified from the brown rot fungus *F. meliae* CFA 2, which was purified 34.18-fold and has a specific activity of 302.90 U/mg. The enzyme exhibits optimal activity at 70°C and a pH of 4.8, with a molecular weight of 37.87 kDa, making it promising for biomass hydrolysis due to its favorable kinetic properties and stability. Its activity is enhanced by Zn^2+^ and K^+^ ions, with a half-life of 11.36 h at 70°C (Patel and Shah, 2021). The cellulolytic-hemicellulolytic enzyme production of *F. meliae* CFA 2, a newly isolated brown rot fungus, was studied. Under solid-state fermentation with wheat bran, it produced 1391.12 U/g of endoglucanase. After statistical optimization, the endoglucanase yield increased by 1.83-fold. Enzymatic saccharification of alkali-treated wheat and rice straw released 190.8 and 318.8 mg/g of reducing sugars, respectively, highlighting its potential for biomass degradation (Patel et al., 2021). Cellulose can be broken down by cellulases for biofuel production. *Fomitopsis meliae* has been cultivated under solid-state fermentation (SSF) on wheat bran, achieving high cellulase yields, particularly CMCase. Using the One-Factor-at-a-Time (OFAT) approach, optimal SSF conditions were determined, including a temperature of 32–36°C, pH 4.0, and a 1:3 moisture ratio. These results highlight *F. meliae* as a promising cellulase producer for industrial applications (Jini and Singh, 2024). Enzymes and proteins isolated from species of *Fomitopsis* along with details on their activities, characteristics, and potential biotechnological applications are presented in **Table 3**.

**Table 3:** Activities, characteristics, and potential applications of enzymes and proteins isolated from species of *Fomitopsis*.

Species	Enzyme/Protein Name	Molecular Mass	Optimal Conditions	Reference
*Fomitopsis meliae* CFA 2	Endoglucanase	37.87 kDa	70°C; pH 4.8	Patel and Shah (2021)
	Endoglucanase (statistically optimized yield)	N/A	N/A	Patel et al. (2021)
*F. palustris*	Malate synthase	520 kDa	Requires Mg²+	Munir et al. (2002)
	Isocitrate lyase	186 kDa	pH 7.0; requires Mg²+ and sulfhydryl	Munir et al. (2002)
	NADP-linked isocitrate dehydrogenase	115 kDa	pH 9.0; requires Mg²+	Yoon et al. (2003)
	Exoglucanases, endoglucanases, β-glucosidase	N/A	pH 4.5; 70°C	Jeong-Jun (2005)
	Glucoamylase	72 kDa	N/A	Yoon et al. (2006)
	Endoglucanase (47 kDa)	47 kDa	N/A	Yoon et al. (2007a)
	Endoglucanase (35 kDa)	35 kDa	N/A	Yoon et al. (2007a)
	Xylanase	43 kDa	pH 4.0–5.0; 70°C	Yoon et al. (2007b)
	Oxaloacetate acetylhydrolase (FpOAH)	N/A	N/A	Hisamori et al. (2013)
	Endoglucanase (cel12)	N/A	N/A	Song et al. (2018)
	β-glucosidase (βGI and βGII)	N/A	pH 2.5; 55°C	Okamoto et al. (2011)
*F. pinicola*	Milk-clotting enzyme (BR)	N/A	35°C; higher heat stability	Nakanishi and Itoh (1969)
	Laccases (FpLcc1 and FpLcc2)	N/A	Low pH; activated by acetic acid	Csarman et al. (2021)
	GH45 endoglucanase	N/A	pH 4	Amengual et al. (2022)
	Cellulases (CMCase, β-glucosidase)	N/A	N/A	Sun et al. (2024)

#### 4.3.2 Fomitopsis palustris

Malate synthase, a key enzyme in the glyoxylate cycle, was purified from *F. palustris*. The enzyme, with a molecular mass of 520 kDa and composed of eight 65-kDa subunits, showed Km values of 45 μM for glyoxylate and 2.2 μM for acetyl-CoA. Its activity was inhibited by oxalate, glycolate, and coenzyme A, with p-chloromercuribenzoate indicating the presence of a sulfhydryl group at the active site. The enzyme required Mg2+ for full activation and stability, becoming inactive without metal ions (Munir et al., 2002). Isocitrate lyase, a key enzyme in the glyoxylate cycle, was purified 76-fold with a 23% yield from *F. palustris* grown on glucose. The enzyme, with a molecular mass of 186 kDa, consists of three 60-kDa subunits. It has a Km of 1.6 mM for isocitrate at pH 7.0 and requires Mg2+ and sulfhydryl compounds for optimal activity. The enzyme is strongly inhibited by oxalate and itaconate, with Ki values of 37 and 68 μM, respectively. These findings suggest that isocitrate lyase plays a regulatory role in fungal growth (Munir et al., 2002). One study investigated the roles of the glyoxylate and tricarboxylic acid cycles in oxalate biosynthesis during fungal development. Enzyme activities were higher during the vegetative stage, with isocitrate lyase contributing to oxalate synthesis early on, while isocitrate dehydrogenase played a key role in glutamate synthesis during *F. palustris* fruiting body formation (Yoon et al., 2002). NADP-linked isocitrate dehydrogenase (EC 1.1.1.42) was purified 672-fold from the copper-tolerant fungus *F. palustris*. The enzyme, with a molecular mass of 115 kDa, consists of two 55-kDa subunits and has Km values of 12.7, 2.9, and 23.9 μM for isocitrate, NADP, and Mg^2+^, respectively, at pH 9.0. Mg^2+^ enhances activity and prevents inactivation. The enzyme is competitively inhibited by 2-oxoglutarate (Ki, 127.0 μM) and strongly inhibited by oxaloacetate and glyoxylate through mixed inhibition (Yoon et al., 2003). *Fomitopsis palustris* has been shown to degrade crystalline cellulose (Avicel) by producing exoglucanases, endoglucanases, and β-glucosidase. After 14 days, the relative crystallinity of Avicel decreased from 83% to 78.5%. The optimal conditions for exoglucanase activity were pH 4.5 and 70°C. Hydrolysis yielded 1.6 mg/mL of glucose after 43 hours, with a cellulose conversion degree of 3.2%, highlighting *F. palustris*’ capability to break down crystalline cellulose (Jeong-Jun, 2005).

*Fomitopsis palustris* produces a 72 kDa extracellular enzyme, identified as a glucoamylase, when grown in cellulose culture with cellobiose. When the enzyme was purified, its amino acid sequence showed high similarity to fungal glycoside hydrolase family 15 glucoamylases. The kinetic activity of the enzyme increased with the substrate’s polymerization, and the glucoamylase gene (*gla*) was cloned via reverse transcriptase PCR (Yoon et al., 2006). Two endoglucanases produced by *F. palustris* were purified, with molecular masses of 47 kDa and 35 kDa. The 47-kDa enzyme resembled fungal glycoside hydrolase family 5, while the 35-kDa enzyme exhibited high cellulase activity despite no homology to known glycosylhydrolases. Both enzymes efficiently degraded Avicel, producing cellobiose as the primary product (Yoon et al., 2007a). An extracellular xylanase from the brown-rot fungus *F. palustris* was purified to a single protein band, showing a molecular mass of approximately 43 kDa on SDS-PAGE. The amino acid sequence of the enzyme indicated significant homology with fungal glycoside hydrolase family 10 xylanases. The optimal activity of the purified xylanase occurred at pH 4.0–5.0 and a temperature of 70^∘^C (Yoon et al., 2007b).

When cDNA and FpTRP26 were isolated from *F. palustris* through yeast transformant screening, they conferred specific resistance to OA. Transformants showed a 65% reduction in OA content when grown with 2 mM OA. FpTRP26 transcript levels increased with OA accumulation and remained high, even in the stationary phase, suggesting that FpTRP26 plays a key role in OA resistance in *F. palustris* (Watanabe et al., 2007). Two acidic β-glucosidases (βGI and βGII) from *Fomitopsis palustris* were purified, showing optimal activity at pH 2.5 and 55°C. Both enzymes effectively hydrolyzed cello-oligosaccharides to release glucose, and *F. palustris* produced high ethanol yields from various sugars, highlighting its potential for bioethanol production (Okamoto et al., 2011). *Fomitopsis palustris* produces two oxalate-generating enzymes, with oxaloacetate acetylhydrolase (FpOAH) playing a dominant role in oxalate biosynthesis. A cloned 1080-bp cDNA confirmed FpOAH activity, and its gene expression was significantly higher than that of glyoxylate dehydrogenase (FpGLOXDH), suggesting FpOAH’s primary role in oxalate production (Hisamori et al., 2013). *Fomitopsis palustris* produces cellulases to degrade cellulose, including a newly identified endoglucanase gene, *cel12*. This gene encodes a protein lacking a cellulose-binding domain but is highly conserved among GH family 12 cellulases. The expression of the gene increases during growth on cellulose, and recombinant *cel12* shows endoglucanase activity on carboxymethyl cellulose, but not crystalline cellulose (Song et al., 2018).

#### 4.3.3 Fomitopsis pinicola

The milk-clotting enzyme (BR) from *F. pinicola* was compared to commercial rennet (HR) for cheese-making. BR had higher activity at 35°C, greater heat stability, and more proteolytic activity than HR. Both enzymes acted similarly on κ-casein, and after 50 minutes, curds produced by BR and HR had the same tension. The study concluded that BR could be a viable substitute for commercial rennet in cheese production (Nakanishi and Itoh, 1969). The solid fermentation product of *F. pinicola* demonstrated notable anti-tumor and anti-oxidation effects in H22 tumor-bearing mice, with high and moderate doses achieving inhibition rates of 66.66% and 64.70%, respectively. Treatment increased serum levels of IL-2 and IFN-γ, reduced MDA levels, and enhanced antioxidant enzyme activities (SOD, CAT, and GSH-PX), highlighting its potential as a therapeutic agent (Sun et al., 2016).

Laccases from *F. pinicola* FP58527 SS1 were detected in the secretome when grown on poplar and spruce wood. Two laccases, FpLcc1 and FpLcc2, were produced and purified for testing. Both showed similar low pH-optima and moderate catalytic efficiency. Notably, FpLcc2 was significantly activated by acetic acid, especially at pH 5.0, suggesting a unique regulatory mechanism in brown rot fungi (Csarman et al., 2021). The heterologous expression and characterization of a GH45 endoglucanase from *F. pinicola* were reported, comparing it with a known GH45 from *Phanerochaete chrysosporium*. Both enzymes, expressed in *Pichia pastoris*, demonstrated an acidophilic nature with an optimal pH of 4 and a preference for β-1,4-glucans. No significant differences were observed between the enzymes from the two fungi (Amengual et al., 2022). Fomitopsis pinicola was identified as the wood decay pathogen affecting Korean pine (*Pinus koraiensis*) in this study through rDNA-ITS analysis and morphological observations (ITS accession number OQ880566.1). The cellulase enzymes, endoglucanase (CMCase) and β-glucosidase, were quantified using the DNS method, and enzyme activity was optimized using a single-factor and orthogonal test. The highest cellulase activity reached 116.94 U/mL under specific conditions, providing a foundation for improving cellulose degradation and advancing biotransformation research by brown-rot fungi (Sun et al., 2024).

## 5 Other bioactive compounds and beneficial medicinal properties

**Figure 4:**
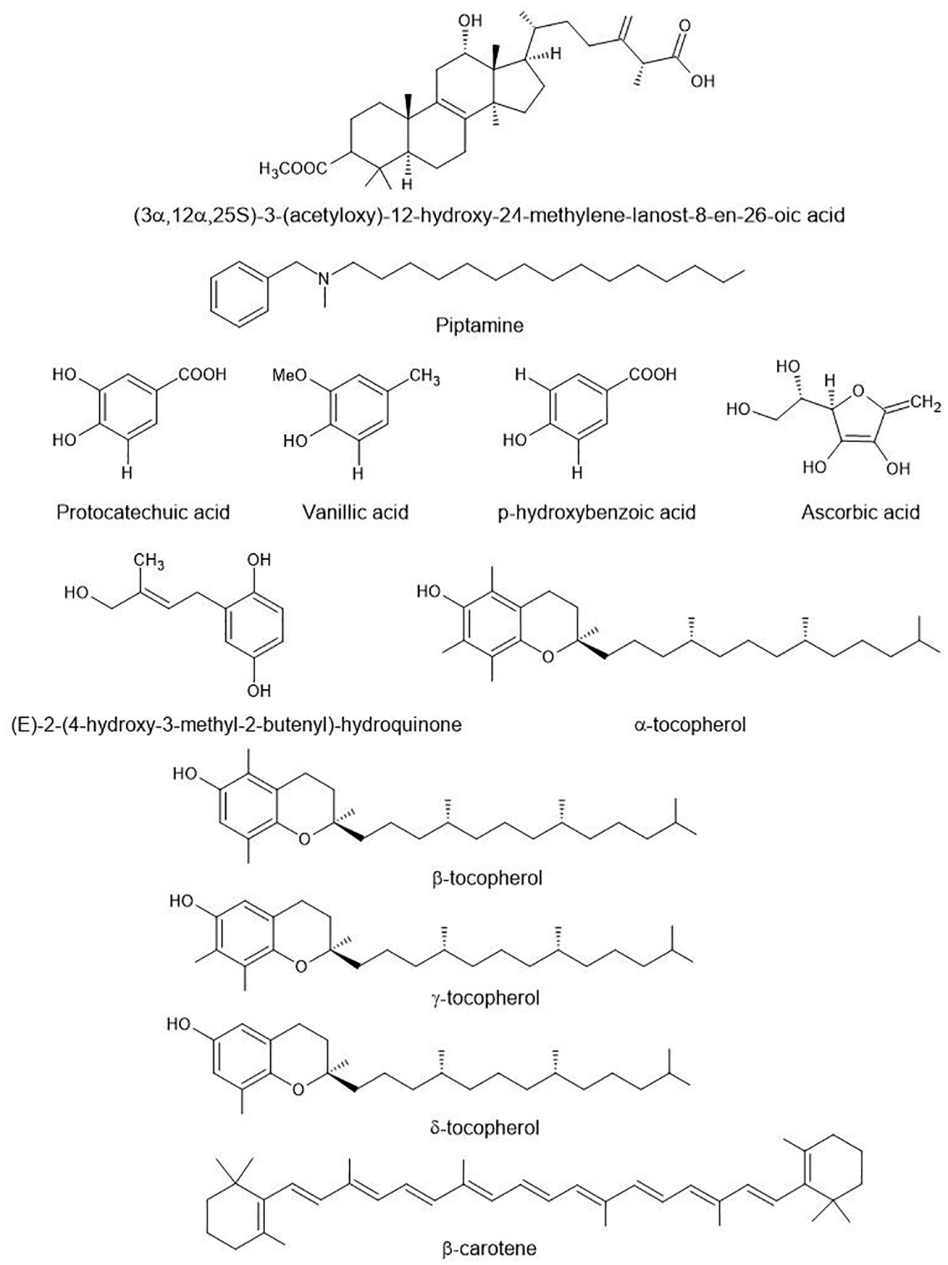
Other bioactive compounds found in species of *Fomitopsis*. These are aromatic amines (piptamine), phenolic acids (protocatechuic acid, vanillic acid, p-Hydroxybenzoic acid), hydroquinone derivatives, and vitamin-related compounds (ascorbic acid, α, β, γ, δ-tocopherol, β-carotene).

In addition to terpenoids, species of *Fomitopsis* are a rich source of diverse bioactive compounds (**Figure 4**) with significant medicinal properties. The following section discusses other bioactive compounds and their beneficial medicinal properties.

### 5.1 Fomitopsis betulina

The methanol and chloroform extracts from fruiting bodies of *F. betulina* exhibited antibacterial properties against *B. subtilis* and *E. coli* (Keller et al., 2002). The ethyl extract fraction derived from dried *F. betulina* fruiting bodies was examined for its impact on various cell lines, including A549, HT-29, murine lung carcinoma, colon adenocarcinoma, and C6 rat glioma. The extract exhibited significant reductions in cell viability, proliferation, and migration among tumor cells, while it also counteracted the stimulating effect of IGF-1, all without causing toxicity to normal cells (Lemieszek et al., 2009). Mycelia of *in vitro*-cultivated *F. betulina* were extracted with ether and ethanol and these extracts showed significant anti-cancer activity, reducing cancer cell viability, slightly inhibiting proliferation, and decreasing tumor cell adhesion. The effects were dependent on the duration and dosage of exposure (Cyranka et al., 2011). An ethyl acetate extract from *Piptoporus betulinus* mycelium increased HaCaT cell viability, reversed G1 cell cycle arrest induced by serum deprivation, and mitigated UV-induced DNA damage by 20% in HaCaT cells. Further, proteome analysis revealed elevated levels of cellular oxidoreductase after treatment with *P. betulinus* (Harms et al., 2013). The hot water extract obtained from the fruiting bodies of *F. betulina* demonstrated a moderate cytotoxic effect, as evidenced by an IC50 value of 0.1 mg/ml against HeLa cells. Furthermore, it significantly inhibited angiotensin-converting enzyme (ACE) activity. These alkali extracts also displayed antioxidant activity in the FRAP assay (Vunduk et al., 2015). Artificially cultivated fruiting bodies of *F. betulina* were used to produce water and ethanol extracts. These extracts exhibited activity against cancer cell lines A549, HT-29, and T47D (Pleszczyńska et al., 2016). In addition, ethanolic extracts of *F. betulina* significantly enhanced granulocyte phagocytosis by 158% (Doskocil et al., 2016). *Fomitopsis betulina* cultivated on agro-industrial by-products produced a pineapple-like aroma. Two compounds, (5E/Z,7E,9)-decatrien-2-ones, were identified as the source of the scent. These compounds were synthesized and confirmed by mass spectrometry, with (5Z,7E,9)-decatrien-2-one having the strongest pineapple-like odor. A specific structure, including 10 carbon atoms, was found to be essential for the aroma (Grosse et al., 2019). *Fomitopsis betulina* shows antibacterial activity against *S. haemoliticus* and *A. baumannii*. Concentrating and drying the cultural liquid improve its antibacterial properties, making it a potential candidate for pharmaceutical applications (Krupodorova et al., 2019).

### 5.2 *F. cajanderi*, *F. feei*, *F. iberica* and *F. meliae*

Hot ethanol and aqueous extracts of *F. cajanderi* were studied for their cytotoxic effects on human cancer cell lines and modulation of TNF secretion. The ethanol extract exhibited concentration-dependent cytotoxicity against MCF7 and U-937 cells, while the aqueous extract was non-cytotoxic to MCF7 cells but inhibited TNF secretion in LPS-stimulated U-937 cells due to its beta-glucan content. These results indicate the potential of *F. cajanderi* extracts as novel adjunctive anti-cancer and anti-inflammatory agents (Wenner et al., 2021). The effect of ten plant oils and eleven mineral chlorides on exopolysaccharide production from *F. feei* was tested in a broth medium. Groundnut oil and sodium chloride positively influenced exopolysaccharide production, providing a scientific foundation for optimizing the extraction of these compounds from *F. feei*, and enhancing its potential medicinal use (Bindu and Charya, 2018). Metabolic profiling of hydroalcoholic and organic extracts from *F. iberica*, *A. biennis*, and *S. hirsutum* mycelia was investigated using NMR methodology. The analysis revealed various amino acids, sugars, organic acids, and fatty acid chains. *Fomitopsis iberica* extracts were notable for the presence of galactose (GABA) and a high amount of ergosterol, highlighting the potential of *Fomitopsis* for developing nutritionally valuable food products (Goppa et al., 2023). Two antibacterial compounds, 5-hydroxymethyl-2-furoic acid methyl ester and 5-hydroxymethyl-2-furancarboxylic acid (HMFCA), were isolated from *F. meliae*. HMFCA demonstrated antibacterial activity against methicillin-susceptible *S, aureus*, while both compounds showed no activity against A549 cancer cells (Srisit et al., 2024).

### 5.3 Fomitopsis officinalis

Several strains of *F. officinalis* show strong antiviral activity, with *F. officinalis I* achieving a Selectivity Index (SI) of >20 against cowpox and *F. officinalis* IV a SI of >29 against the *Vaccinia* virus. The variation in potency underscores the importance of preserving *F. officinalis* biodiversity, which is threatened by habitat destruction. Collecting and studying more strains is vital for developing medicines against pox viruses. Research is ongoing to identify the active antiviral agents, their modes of action, and their impact on immune defense (Stamets, 2005). A family 12 endoglucanase (EG-II) with a molecular mass of 23,926 Da was purified and characterized from the brown-rot basidiomycete *F. palustris*. EG-II is believed to play a role in wood degradation by loosening the polysaccharide network in cell walls through the disentangling of hemicelluloses associated with cellulose (Shimokawa et al., 2008). Agaricinic acid, extracted from *F. officinalis* carpophores using ethanol, was identified through NMR spectroscopy and comparison to a standard sample. The extraction process involved ethanol, followed by purification with ether. The analysis revealed the presence of hydroxyl and various carboxy groups (Airapetova et al., 2010).

*Fomitopsis officinalis* is reported to exhibit broad-spectrum antibacterial and antiviral effects against various pathogens, including *Mycobacterium tuberculosis*, *Yersinia pseudotuberculosis*, *S. aureus*, and the Ortopox virus. Chlorinated coumarins from mycelia and lanostane triterpenoids from basidiomes have been linked to antiviral-antibacterial and trypanocidal activities, respectively. While there is significant *in vitro* potential with crude extracts, standardization remains a challenge (Sidorenko, 2009; Girometta, 2019). *Fomitopsis officinalis* EtOH extract produced two new chlorinated coumarins, identified through spectroscopy and chemical synthesis. Analogues were also synthesized. These compounds had limited antimicrobial activity, with the lowest MICs against *M. tuberculosis* complex (Hwang et al., 2013). Mycelium and fruiting bodies of *F. officinalis* contain various compounds such as L-tryptophan, phenolics (e.g., p-hydroxybenzoic acid, gallic acid), sterols (including ergosterol and ergosterol peroxide), and trace elements. These extracts showed antioxidant and growth-inhibitory effects on cancer cell lines like A549 lung cancer, DU145 prostate cancer, and A375 melanoma cells (Fijałkowska et al., 2020). The effects of adding zinc and magnesium salts to the culture medium in 10-L bioreactors were examined in this study. Results showed that mycelium grown on sulfate-enriched medium had higher levels of these minerals compared to the fruiting bodies. The enrichment increased the bioavailability of bioelements and organic compounds (indole, phenolic compounds, and L-phenylalanine), indicating that the method effectively produces fortified mycelium as a natural therapeutic material (Fijałkowska et al., 2021). The antimicrobial effects of *F. officinalis* were examined through culture fluid and mycelial mass extracts from various strains. The extracts exhibited high activity against *S. aureus*, particularly strains IBK-5004 and IBK-2498, and moderate activity against *Klebsiella pneumoniae*. However, no activity was observed against *Escherichia coli*, *Pseudomonas aeruginosa*, or *B. subtilis* (Mykchaylova and Poyedіnok, 2021). The biological activity of *F. officinalis* was evaluated for its antioxidant and anticancer effects using six extracts against hepatocellular carcinoma cells. All extracts showed antioxidant and anticancer potential. The chloroformic extract (Fo3) notably induced apoptosis, activated the G2/M cell cycle phase and selectively influenced NF-kB proteins. This highlights Fo3’s potential as a natural antitumor agent, warranting further research for its use in cancer treatment (Altannavch et al., 2022).

A study investigates the metabolic differences between the cap (median and apical parts) and hymenium of *F. officinalis*. Chromatographic analysis revealed that the apical part is richest in phenolic compounds. These extracts showed strong antifungal, antibacterial, and antiradical activity, especially against Gram+ bacteria and dermatophytic species, with MIC values below 100 µg/mL, suggesting *F. officinalis* as a valuable source for antioxidant and antimicrobial food supplements (Flores et al., 2023).

### 5.4 Fomitopsis palustris

*Fomitopsis palustris* primarily converts glucose to oxalic acid rather than fully oxidizing it through the TCA cycle. Key enzymes, including isocitrate lyase and oxaloacetase, connect the TCA and glyoxylate cycles to facilitate oxalate production, while malate dehydrogenase plays a crucial role in generating NADH. This process allows the fungus to obtain biochemical energy (Munir et al., 2001). A novel enzyme, FpPG28A, was isolated from *F. palustris*. It collaborates with oxalic acid to break down wood pectin. FpPG28A doesn’t affect esterified pectin, suggesting the involvement of a pectin esterase. This enzyme operates optimally at 60°C and pH 5.0 and efficiently degrades pectin in the presence of oxalate. Oxalate also enhances its thermostability at pH 3.0, highlighting its role in wood pectin degradation (Tanaka et al., 2018).

### 5.5 Fomitopsis pinicola

A cerebroside fraction was extracted from *F. pinicola* fruiting bodies, with the main cerebroside determined to have the structure (4E,8E,2S,3R,2’R)-N-2’-hydroxypalmityl-1-O-α-D-glucopyranosyl-9-methyl-4,8-sphingadienine (Striegler and Haslinger, 1996). N-hexane and methanol extracts from *F. pinicola* identified six new lanostanoid derivatives, confirmed using mass spectrometry and NMR (Rösecke and König, 1999). The fruiting body of *F. pinicola* has been analyzed for nutritional components, revealing high fiber (43%) and carbohydrate (23%) contents, along with 12% amino acids. Glutamate was the most abundant amino acid, and vitamin C was the dominant vitamin at 276 mg/100 g dry mushroom. Key minerals included potassium (16.86 mg/g) and calcium (16.19 mg/100 g) (Ding et al., 2006). Methanol extracts from *F. pinicola* showed strong inhibition against *Helicobacter pylori*, with maximum activity after 8 days of fermentation and a MIC of 0.25 mg. The most active fraction, Fp-T3, identified as an aminosugar, displayed significant inhibitory activity (14.4 mm ID) against *H. pylori* (Lee et al., 2006). The hypoglycemic effect of *F. pinicola* extracts was tested in alloxan-induced diabetic rats. Rats given the hot-water extract showed a significant decrease in blood glucose levels, while those given the ethanol extract experienced a minimal reduction compared to the control group with 600 mg/dl (Shin et al., 2007). *Fomitopsis pinicola* extracts possess significant anti-oxidant and anti-tumor activities comparable to the conventional extracts. However, further studies are necessary to elucidate the relationship between antioxidant and antitumor activities and the pharmacological activity of the *F. pinicola* extract. Also, as the growth of *F. pinicola* in nature is very slow. As such, isolation and artificial culture may be required for further progress in the mass production of this compound (Choi et al., 2007).

The alkali extract (AE) from *F. pinicola* significantly lowered blood glucose levels and improved weight gain in streptozotocin (STZ)-induced diabetic rats while also restoring serum insulin levels and reducing pancreatic damage. This study is the first to demonstrate the antihyperglycemic effects of *F. pinicola* in this model, suggesting its components may enhance insulin secretion during recovery or protect pancreatic tissue from STZ-induced damage (Lee S. et al., 2008). *Fomitopsis pinicola* extracts showed potential antifungal activities against *Fusarium inflexum* and *F. heterosporium* (Guler et al., 2009). *Fomitopsis pinicola* showed high cellobiohydrolase (CBH) activity. The purified CBH, a 64 kDa monomer, is highly stable at high temperatures and displays efficient catalysis. It differs from other CBHs due to its exceptional catalytic efficiency and thermostability (Shin et al., 2010a). Extracellular xylanase from *F. pinicola* was purified using chromatography techniques. The enzyme, with a molecular weight of 58 kDa, showed optimal activity at 71°C and pH 4.6, with a half-life of 33 hours at 70°C. It had a catalytic efficiency of kcat = 77.4 s^−1^ and kcat/km = 22.7 mg/ml/s. The amino acid sequence revealed homology with GH family 10 hydrolases, confirming *F. pinicola* as a member of this family (Shin et al., 2010b). The anti-tumor activities of *F. pinicola* extract (FP-I) were studied *in vitro* and *in vivo*. FP-I inhibited the proliferation of mouse hepatocellular carcinoma (H22) and sarcoma (S180) cells. In an S180 mouse model, FP-I significantly reduced tumor growth and improved immune function indicators, such as lymphocyte proportion and thymus index. The extract also induced apoptosis in tumor cells. These results suggest that *F. pinicola* extract possesses anti-tumor effects related to immune enhancement and apoptosis induction (Xiao et al., 2011). Chloroform, petroleum, water extracts, and Compound A were studied for antitumor activity. The chloroform extract boosted immune function, increasing the spleen index and IL-2 levels. One compound (Compound A) showed strong antitumor effects, with a 52.31% inhibition rate *in vivo* and significant inhibition of breast (MCF-7) and liver (SMMC-7721) cancer cells *in vitro*, suggesting it is the main antitumor agent in *F. pinicola* (Sun et al., 2012).

The effect of *F. pinicola* chloroform extract (FPKc) on SW-480 cancer cells involved inhibiting cell viability, reducing migration, and inducing ROS-mediated apoptosis. FPKc also caused G1 phase arrest and decreased MMP-2 and MMP-9 expression. Ergosterol (ES), a major component of FPKc, demonstrated similar effects, contributing to its anti-cancer activity (Wang et al., 2014). *Fomitopsis pinicola* mycelium extract produces extracellular antifungal metabolites and possesses antifungal activity against potentially pathogenic filamentous fungi. Further research on the antifungal activity of *F. pinicola* mycelial extract may aid in the development of antimycotic biotechnological products from this mushroom (Badalyan et al., 2014). Research indicated that *F. pinicola* ethanol extract possesses anti-cancer properties against S-180 malignant cells, both in laboratory and animal tests. It was observed to induce apoptosis in advanced stages of lung, colorectal, breast, and hepatoma cancer cells (Wu et al., 2014). *Fomitopsis pinicola* extracts were evaluated for their antioxidant properties, including DPPH radical scavenging activities and their impact on important antioxidant enzymes such as SOD, CAT, and GPx. These enzymes help protect against oxidative stress and related diseases like Alzheimer’s, Parkinson’s, cancer, and aging (Onar et al., 2016). AVA, a formulation containing *F. pinicola* Jeseng extract, significantly combats obesity and nonalcoholic fatty liver disease (NAFLD) in high-fat diet-induced obese mice. The anti-obesity effects may result from inhibiting specific genes and cholesterol synthesis. FAVA could be a promising dietary supplement to prevent obesity and NAFLD (Jung et al., 2016). The antioxidant and heavy metal content of *F. pinicola* from Kazdağı and Çınarcık in Turkey were examined. Higher oxidative stress index (OSI) and iron levels were found in Çınarcık samples. These fungi may serve as antioxidant sources, but elevated heavy metals could increase oxidative stress (Sevindik et al., 2017). The chloroform extract derived from *F. pinicola* effectively curbed the proliferation of S180 tumor cells and contributed to the extended survival of mice. In a laboratory setting, it was evident that FPKc induced apoptosis in S180 tumor cells and brought about cell cycle arrest, most likely through the mitochondrial pathway (Gao et al., 2017).

The antitumor effects of *F. pinicola* chloroform extract (FPKc) was investigated on S180 tumor cells, revealing its active components and significant inhibitory effects on cell proliferation, leading to apoptosis and cell cycle arrest. *In vivo*, FPKc inhibited tumor growth and extended the survival of tumor-bearing mice while sparing normal cells. The findings suggest that FPKc induces tumor cell apoptosis primarily through mitochondrial pathways. Neuroprotective effects of water extract from *F. pinicola* were assessed in mesencephalic dopaminergic cells exposed to MPP+. The extract improved survival and neurite growth of TH-immunoreactive neurons while enhancing mitochondrial respiratory chain complex I activity and reducing apoptosis rates at doses of 50 and 25 μg/mL. These findings indicate that *F. pinicola* protects dopaminergic cells from MPP+-induced damage (Guo and Rausch, 2018). Comparing mycelium and fruiting body extracts of *Fomitopsis*, the mycelium extract demonstrated high cytotoxicity to prostate cancer cells, while both extracts displayed potential anti-inflammatory effects. These findings indicate their biotechnological potential as sources of bioactive compounds (Sułkowska-Ziaja et al., 2018). The *F. pinicola* extract displayed weak antioxidant properties but effectively hindered the growth of human tumor cells in a dose-dependent manner. It induced apoptosis in one cell line but showed toxicity in another. No DNA damage was observed in normal human leukocytes exposed to the extract. The extract exhibited variable antifungal effects against pathogenic fungi. Overall, the extract demonstrated potent antimicrobial and chemo-preventive activities but had limited antioxidant capabilities (Angelini et al., 2018). The anti-inflammatory, analgesic, and antipyretic effects of *F. pinicola* fruiting body extracts were tested in mice. Water and n-butanol extracts had the strongest effects, reducing capillary permeability, pain, and fever. Methylene chloride and petroleum ether extracts reduced ear swelling and oxidative stress. The active compounds are concentrated in the highly polar extracts, showing significant anti-inflammatory and pain-relief properties (Zhao and Bao, 2019).

Ethyl acetate and methanolic extract of *F. pinicola* extracts showed significant antimicrobial activity against a broad spectrum of microbes (Pala et al., 2019). Neuroprotective effects of the water extract of *F. pinicola* were investigated using primary dopaminergic cell cultures from embryonic mouse mesencephala subjected to MPP+ toxicity. The extract demonstrated significant protection against dopaminergic neuron degeneration, exhibiting antioxidant and anti-inflammatory activities. The mechanism underlying its neuroprotective effect is likely related to inhibiting mitochondrial oxidative stress (Guo and Rausch, 2019). Treatment with *F. pinicola* extract did not exhibit a statistically significant influence on PC3 prostate cancer tumor progression in mice. However, it demonstrated notable growth-inhibitory properties *in vitro* using the same cell line. This underscores the continued potential of *F. pinicola* as a valuable reservoir of bioactive compounds with anti-cancer properties (Kao et al., 2020). The growth-inhibitory potential of eight wild British Columbian mushrooms, including *F. pinicola, Phaeolus schweinitzii*, and *Phaeolus* sp., was investigated in this thesis. Of the 28 crude extracts tested, 15 demonstrated significant inhibitory activity. Hispidin, a known anti-cancer compound, was purified from *Phaeolus* sp. through liquid-liquid extraction and HPLC-MS, while another compound with a mass-to-charge ratio of 283.2 was detected. This research provides a foundation for further studies on these species, including *F. pinicola*, as sources of bioactive compounds (Da, 2020).

Ethyl acetate extract (EAE) of *F. pinicola* exhibited significant cytotoxicity (IC50 of 100 µg/mL), inhibited tumor growth (at 500 mg/kg), antiangiogenesis, and halted cell cycle progression at the G1 phase. The chemical analysis identified 11-α-acetoxykhivorin as the major active component. This suggests that *F. pinicola* EAE has potent antineoplastic effects, possibly due to its key chemical constituents (Ravikumar et al., 2021). The antibacterial activity of *F. pinicola* BCC58 was evaluated under different cultivation conditions. Xylose, glucose, and mandarin squeeze showed the highest inhibition of *S. aureus* and *E. coli*. Supplementing with KNO3 or yeast extract enhanced ABA. Ethanolic extracts from biomass and culture liquid had the strongest ABA, especially against *E. coli* with an MIC of 0.5 mg/mL, while hot water and ethyl acetate extracts showed lower activity (Metreveli et al., 2021). The antioxidant and cytoprotective activities of ethanol extracts from *F. pinicola* (FPE) were evaluated. UPLC-MS/MS analysis identified 14 bioactive compounds in FPE, including 8 triterpenoids, 4 triterpene glycosides, 1 lanosterol, and 1 lanostanoid. FPE demonstrated potent *in vitro* antioxidative effects, with a DPPH scavenging rate of 91.76% at 1.4 mg/mL and an ABTS radical scavenging rate of 100% at 0.6 mg/mL. In addition, FPE effectively protected against AAPH-induced oxidative damage and inhibited cell aging in cytoprotection assays (Li et al., 2022). The potential benefits of the chloroform extract of *F. pinicola* (FPKc) on ulcerative colitis (UC) were explored in a study using a DSS-induced UC mouse model. Treatment with FPKc improved symptoms such as hematochezia and weight loss, reduced disease activity and colonic damage indices, and enhanced colon tissue structure. FPKc also lowered pro-inflammatory cytokines (IL-6, IL-8) and reduced AST and ALT levels, indicating its protective effects may be linked to immune regulation and inflammation reduction (Cheng et al., 2023). *Fomitopsis pinicola* extract (EMFP) showed strong antioxidant and anticancer properties. EMFP effectively scavenged free radicals, protected against protein oxidation, and inhibited HepG2 cell proliferation by increasing ROS, depleting mitochondrial membrane potential, and inducing apoptosis. It also altered oxidative stress markers and contained phenolic compounds and triterpenes, contributing to its therapeutic potential (Zhang et al., 2023).

Bioactive metabolite production can be enhanced by optimizing *F. pinicola* cultivation conditions. The highest biomass (8.5 g/L) was achieved at 20°C, while maximum antioxidant activity (78.2%) occurred at 30°C. Xylose and peptone promoted phenol synthesis, whereas galactose and yeast extract supported biomass growth. The fungus adapted to pH 2.5–7.5, with shaking conditions maximizing phenol yield (21.44 mg GAE/g). These findings highlight strategies for improving fungal cultivation to enhance bioactive compound production (Krupodorova et al., 2024). The antimicrobial activity of 14 dikaryotic strains of *F. pinicola* isolated from various trees in Russia, France, and Italy was evaluated against dermatophytes, species of *Penicillium*, and both Gram-negative and Gram-positive bacteria. Cultural broth samples demonstrated stronger antifungal and antibacterial effects than mycelial extracts, indicating the potential of *F. pinicola* as a source of antimicrobial compounds for future biotech applications. Further studies are needed to elucidate the underlying mechanisms of its antimicrobial effects (Badalyan et al., 2024). **Table 4** summarizes the chemical analyses, bioactivities, and potential medicinal applications of various *Fomitopsis* species, while **Table 5** highlights the diverse bioactivities and chemical compounds species of *F. pinicola*.

**Table 4:** Chemical analyses, bioactivities, and potential medicinal applications of various species of *Fomitopsis*.

Species	Extract/Compound	Activity	Target/Effects	Reference
*Fomitopsis betulina*	Concentrated, dried cultural liquid	Antibacterial	*S. haemoliticus*, *A. baumannii*	Krupodorova et al. (2019)
	Methanol, chloroform extracts (fruiting bodies)	Antibacterial	*B. subtilis, E. coli*	Keller et al. (2002)
	Hot water extract (fruiting bodies)	Cytotoxic, ACE inhibition, Antioxidant	HeLa cells (IC50 0.1 mg/ml), ACE activity inhibition, FRAP assay	Vunduk et al. (2015)
	Ethyl extract fraction (fruiting bodies)	Anti-cancer	A549, HT-29, murine lung carcinoma, colon adenocarcinoma, C6 rat glioma	Lemieszek et al. (2009)
	Ether, ethanol extracts (mycelia)	Anti-cancer	Cancer cell viability, proliferation inhibition, tumor cell adhesion reduction	Cyranka et al. (2011)
	Water, ethanol extracts (cultivated fruiting bodies)	Anti-cancer	A549, HT-29, T47D	Pleszczyńska et al. (2016)
	Ethanolic extracts	Immunomodulatory	Granulocyte phagocytosis (158%)	Doskocil et al. (2016)
	Aromatic compounds (decatrien-2-ones)	Aroma (Pineapple-like)	Pineapple-like odor	Grosse et al. (2019)
*F. castaneus*	Exopolysaccharides (EPS)	Prebiotic, SCFA production	Increased SCFA production in simulated human intestinal environment	
*F. cajanderi*	Hot ethanol and aqueous extracts	Cytotoxic, anti-inflammatory	MCF7, U-937 cells, TNF secretion inhibition	Wenner et al. (2021)

**Table 5:** Diverse bioactivities and chemical compounds of *Fomitopsis pinicola*.

Source/Type of *F. pinicola*	Type of Extract/Compound	Administration Route	Specific Effects	Reference
Fruiting body	Chloroform extract (FPKc) Chloroform extract (FPKc)	Oral (*in vivo*, mice)	Inhibited tumor growth, extended survival, apoptosis via mitochondrial pathway in S180 tumor cells	Gao et al. (2017)
		Oral (*in vivo, mice)*	Inhibited S180 tumor cell proliferation, apoptosis, and cell cycle arrest	
		Oral (*in vivo*)	Improved symptoms of ulcerative colitis in DSS-induced mouse model	Cheng et al. (2023)
	Chloroform extract and Compound A	*In vivo*, *in vitro*	Antitumor, increased immune function, spleen index, IL-2 levels, inhibited breast and liver cancer cells	Sun et al. (2012)
	Water extract	*In vitro*	Neuroprotective, improved mitochondrial function in mesencephalic dopaminergic cells	Guo and Rausch (2018)
		*In vitro*	Neuroprotective effects against dopaminergic neuron degeneration	Guo and Rausch (2019)
	Water, n-butanol, methylene chloride, petroleum ether extracts	*In vivo* (mice)	Anti-inflammatory, analgesic, antipyretic effects	Zhao and Bao (2019)
	Ethanol extract	*In vivo, in vitro*	Induced apoptosis in cancer cells, anti-cancer effects	Wu et al. (2014)
		*In vitro*	Antioxidant, cytoprotective, identified 14 bioactive compounds	Li et al. (2022)
	Ethyl acetate extract (EAE)	Oral (*in vivo*)	Cytotoxicity, tumor growth inhibition, antiangiogenesis, cell cycle arrest	Ravikumar et al. (2021)
		*In vitro*	Antimicrobial activity against a broad spectrum of microbes	Pala et al. (2019)
	Ethyl acetate, methanolic extract	*In vitro*	Antimicrobial activity against a broad spectrum of microbes	Pala et al. (2019)
	Water, ethyl acetate extracts	*In vitro*	Antifungal activities against Fusarium species	Guler et al. (2009)
	Various extracts (DPPH radical scavenging)	*In vitro*	Antioxidant properties, impact on SOD, CAT, GPx enzymes	Onar et al. (2016)
	Cellobiohydrolase (CBH)	*In vitro*	High catalytic efficiency, thermostable enzyme	Shin et al. (2010a)
	Extracellular xylanase	*In vitro*	Optimal activity at 71°C, high stability	Shin et al. (2010b)
	Alkali extract (AE)	Oral (*in vivo*)	Reduced blood glucose levels, and improved serum insulin in diabetic rats	Lee S. et al. (2008)
	Ethanolic, water, and ethyl acetate extracts	*In vitro*	Antibacterial activity against *S. aureus and E. coli*	Metreveli et al. (2021)
	Various extracts (antioxidant, antimicrobial)	*In vitro*	Antimicrobial and antifungal activities against various pathogens	Badalyan et al. (2024)
Jeseng formulation	Jeseng extract (AVA formulation)	Oral (*in vivo*)	Anti-obesity, reduced NAFLD symptoms in high-fat diet mice	Jung et al. (2016)
Mycelium	Mycelium extract	*In vitro*	Antifungal activity, potential development for antimycotic products	Badalyan et al. (2014)
	Ethyl acetate extract	*In vitro*	Increased HaCaT cell viability, reduced UV-induced DNA damage	Harms et al. (2013)

## 6 Clinical trials and patents

Despite these promising bioactivities, human clinical trials on *Fomitopsis* remain scarce. The MACH19 study (NCT04667247) is currently evaluating the safety and feasibility of using FoTv—a combination of *F. officinalis* and *T. versicolor*—for treating mild-to-moderate COVID-19 in outpatients. This randomized, double-blind, placebo-controlled trial assesses safety, disease progression, and immune response markers. Similarly, another MACH19 study (NCT04951336) investigates the potential of FoTv mushrooms to enhance immune response and mitigate vaccine-related side effects in individuals receiving the COVID-19 vaccine. This study follows a similar design, focusing on immune parameters such as antibody titers. Both trials are ongoing, with completion expected by December 2024, but no published findings are available yet (Saxe, 2021a, 2021b).

A patent was disclosed for *F. pinicola* extracts, including both fruit body and mycelial extracts, which demonstrated the inhibition of renal and retinal aldose reductase activity. These extracts also reduced triglycerides, total cholesterol, and LDL cholesterol, suggesting their potential for developing functional foods aimed at preventing diabetes complications and managing diabetes-induced hyperlipidemia (Oh et al., 2007). A patent was granted for active ingredients from *Polyporus officinalis* (= *F. officinalis*), primarily eburicoic acid, dehydroeburicoic acid, and Versisponic acid D, with over 50% total triterpenoid acid. A purification method using macroporous resin and a medicinal preparation were also provided. Animal studies showed efficacy in preventing and treating tumors, particularly liver, stomach, and colon cancer (Zhou, 2012). A patent has been issued for dehydrosulfurenic acid, a compound unique to *Fomes officinalis* (=*F. officinalis*), as a potential treatment for ischemic stroke. When administered at 50 mg/kg just 10 minutes before an ischemic event, it significantly alleviated motor impairments and neuronal damage (Simi and Prisco, 2018). A patent by Sidorenko and Buzoleva (2012) was based on *F. officinalis* mycelia-based preparations against *pseudotuberculosis*, owing to their inhibitory effect on *Y. pseudotuberculosis* (Sidorenko and Buzoleva 2012).

Stamets has outlined several practical applications utilizing the antimicrobial properties of fungi, with a focus on the mycelial phase (Stamets, 2011). While the research includes numerous fungal species, *F. officinalis* is consistently emphasized for its distinct characteristics (Stamets, 2011). In one study on the antiviral properties of medicinal mushrooms, *F. officinalis* demonstrated activity against Cowpox and Vaccinia viruses in human foreskin fibroblast (HFF) cells (Stamets, 2011). Notably, the extract was found to be devoid of agaric acid, leaving its potential bioactivity unclear (Stamets, 2011). Furthermore, in research exploring both antiviral and antibacterial effects, the extract led to a significant reduction in colony-forming units (CFUs) of *E. coli* and *S. aureus* (Stamets, 2014). A 1–2% extract from *F. officinalis* demonstrated 50% inhibition of virus-induced cellular damage (EC50), with a 1:106 diluted crude extract still effective against influenza A, B, and herpes. The extract showed high selectivity and significant inhibition against *M. tuberculosis* (Stamets, 2018). The patent CA 2980173 covers the antiviral properties of medicinal mushrooms containing phenyl carboxylate/acrylate compounds. It includes a diverse group of medicinal mushrooms, potentially encompassing *F. officinalis*, which has been recognized for its antimicrobial and antiviral properties in other studies and patents by Stamets. The invention explores fungal-derived compounds for antiviral applications, emphasizing their therapeutic potential against various viral pathogens (Stamets, 2021).

## 7 *Fomitopsis* based products and their market potential

**Figure 5:**
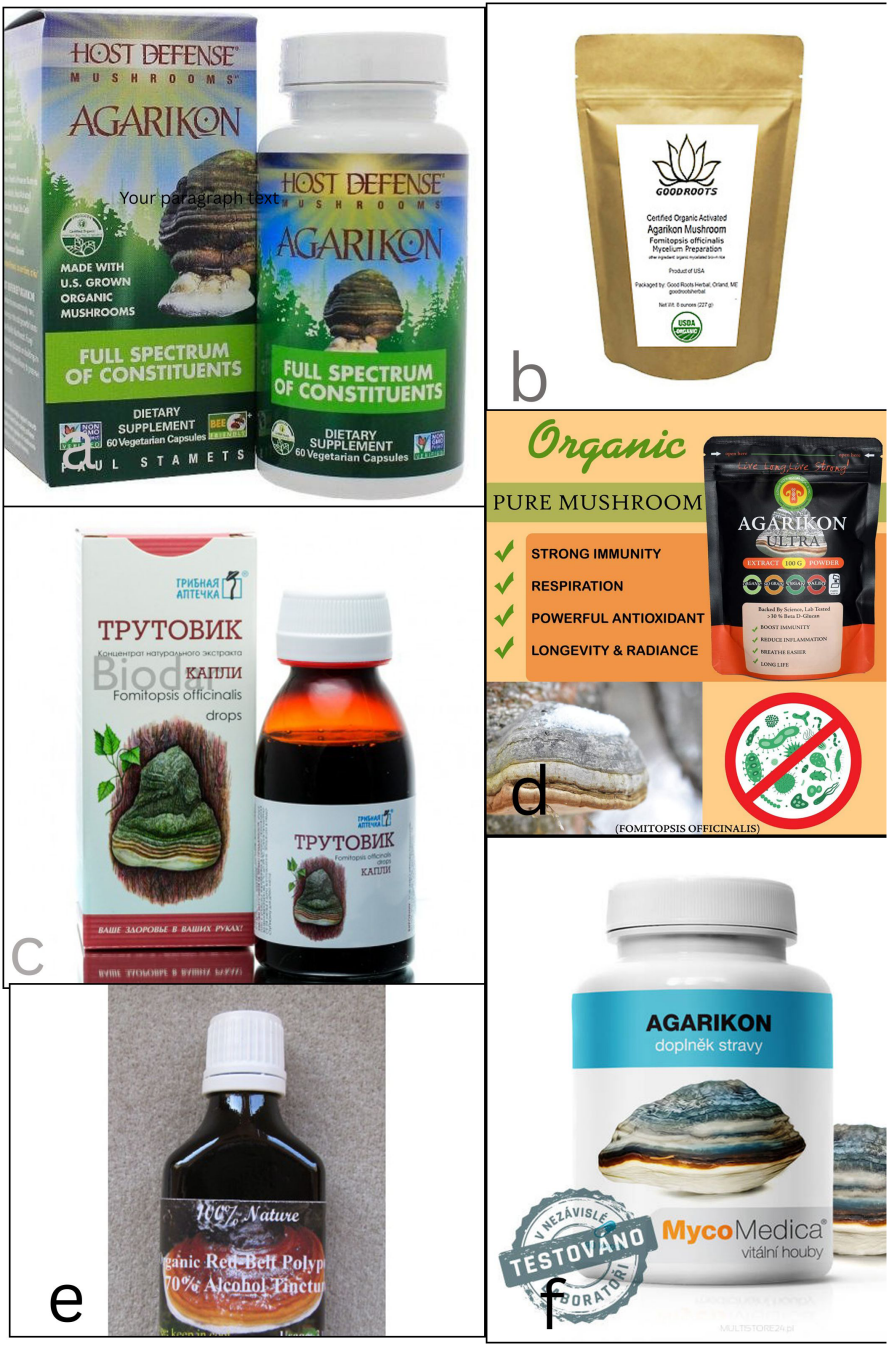
*Fomitopsis* based products. **(a)** Agarikon powder capsules (www.Hostdefense.com), **(b)** Agarikon mushroom powder (www.goodrootsllc.com) **(c)** *Fomitopsis officinalis* drops (dropsukrainashop.com) **(d)** AGARIKON Ultra (https://longevitybotanicals.com) **(e)** Red belt tincture (https://www.herbal-goods.com/) **(f)** Mycomedia Agarikon capsules (www.mycomedica.eu).

The market potential for *Fomitopsis*-based products, particularly *F. officinalis* (Agarikon), is increasing due to growing interest in natural health supplements and medicinal mushrooms (**Figure 5**). This species has been recognized for its potential antiviral, anti-inflammatory, and immune-boosting properties, making it a valuable ingredient in nutraceuticals, pharmaceuticals, and functional foods (**Table 6**). Products such as extracts, tinctures, capsules, and powders are in demand, with potential expansion into skincare and therapeutic applications (Out-grow.com). However, challenges such as slow natural growth and limited large-scale cultivation methods hinder commercial scalability. Efforts to improve cultivation techniques, such as liquid cultures and controlled growth environments, are being explored to enhance production and meet increasing demand (Out-grow.com). In addition, the commercialization of medicinal mushrooms is gaining traction in global markets, further supporting the economic potential of *Fomitopsis*-derived products (Businesswire.com). The growing demand for natural and sustainable health solutions has also driven research into optimizing extraction methods and bioactive compound yields from *Fomitopsis*. This has opened new opportunities for its use in personalized medicine and integrative healthcare approaches. Furthermore, the integration of advanced biotechnological tools, such as genetic engineering and metabolomics, is expected to accelerate the development of high-quality, standardized products.

**Table 6:** A comparative analysis of *Fomitopsis* based-products based on form, key ingredients, benefits, and usage instructions.

Product Name	Form	Manufacturer/Source	Key Ingredients	Benefits	Usage Instructions
AGARIKON Ultra	Tincture	Longevity Botanicals	Agarikon mushroom extract, organic alcohol	Supports immune health, antioxidant properties	Take 1-2 mL (approx. 30-60 drops) daily, diluted in water or juice.
Agarikon Defense Formula	Capsules	Om Mushrooms	Agarikon mushroom, organic rice flour	Boosts immune system, promotes respiratory health	Take 2 capsules daily with water, preferably with meals.
Agarikon Dual Extract	Liquid Extract	Real Mushrooms	Dual-extracted Agarikon mushroom	Enhances immune support, anti-inflammatory properties	Take 1 mL (approx. 30 drops) daily, mixed with water or tea.
Agarikon Immune Boost	Tea Bags	Mushroom Wisdom	Agarikon mushroom, organic herbs	Supports immune function, provides antioxidants	Steep 1 tea bag in hot water for 5-10 minutes, drink daily.
Agarikon Mushroom Chocolates	Chocolate Bars	Honey Pacifica	Agarikon extract, dark chocolate, honey	Immune support, delicious and convenient	Enjoy 1-2 squares daily as a treat or snack.
Agarikon Mushroom Coffee	Instant Coffee Mix	Four Sigmatic	Agarikon extract, organic coffee	Boosts energy, supports immune health	Mix 1 packet with hot water, stir, and enjoy daily.
Agarikon Mushroom Elixir	Liquid Elixir	Pure Mushrooms	Agarikon extract, organic honey, herbs	Immune support, promotes overall wellness	Take 1-2 teaspoons daily, directly or mixed into beverages.
Agarikon Mushroom Extract	Powder Extract	New Earth Organics	Pure Agarikon mushroom extract	Supports immune health, antioxidant properties	Mix 1/2 tsp in water, juice, or smoothies daily.
Agarikon Mushroom Gummies	Gummies	FreshCap Mushrooms	Agarikon extract, organic cane sugar, pectin	Immune support, easy and tasty	Chew 2 gummies daily as a dietary supplement.
Agarikon Mushroom Powder	Powder	Good Roots LLC	Pure Agarikon mushroom powder	Supports immune and respiratory health	Mix 1 tsp into beverages, soups, or smoothies daily.
Agarikon Mushroom Syrup	Syrup	Honey Pacifica	Agarikon extract, raw honey, herbs	Immune support, soothing for throat and respiratory health	Take 1-2 teaspoons daily, directly or mixed into tea.
Agarikon Mushroom Tonic	Liquid Tonic	Shaman Shack Botanicals	Agarikon extract, adaptogenic herbs	Boosts immunity, supports stress relief	Take 1-2 mL (approx. 30-60 drops) daily, diluted in water or juice.
Agarikon Powder Capsules	Capsules	Host Defense	Organic Agarikon mushroom powder	Promotes immune system, supports respiratory health	Take 2 capsules daily with water.
Agarikon Powder Extract	Powder Extract	Fungi Family Farm	Agarikon mushroom extract, organic	Immune support, antioxidant-rich	Mix 1/2 tsp in water, juice, or smoothies daily.
Agarikon Mushroom Complex	Powder Blend	Terrasoul Superfoods	Agarikon extract, other medicinal mushrooms	Comprehensive immune support, adaptogenic benefits	Mix 1 tsp into beverages, soups, or smoothies daily.
*Fomitopsis officinalis* Drops	Liquid Drops	Drops Ukraina Shop	Agarikon mushroom extract, purified water	Immune support, traditional herbal remedy	Take 10-15 drops daily, diluted in water or tea.
Mycomedica Agarikon Capsules	Capsules	Mycomedica	Agarikon mushroom extract, organic rice flour	Supports immune health, promotes vitality	Take 1-2 capsules daily with water.
Red Belt Tincture	Tincture	Herbal Goods	Agarikon extract, organic alcohol	Immune support, anti-inflammatory properties	Take 1 mL (approx. 30 drops) daily, diluted in water or juice.

## 8 Biotechnological applications

### 8.1 Bioremediation

Recently, various synthetic chemicals, including a wide range of solvents, pesticides, and plasticizers, have seen extensive use. Chemical pesticides, crucial for safeguarding crops, represent a prominent example, with the commonly used 1, 1, 1-trichloro-2, 2-bis(4-chlorophenyl) ethane, referred to as DDT, being a notable illustration (Hai et al., 2012). The presence of chlorine atoms in DDT contributes to its high toxicity towards complex organisms and is attributed to its partial solubility and propensity for partitioning into the lipophilic phase (Sudharshan et al., 2012). Its notable persistence and toxicity characteristics have led to its classification as a primary environmental pollutant by the United States Environmental Protection Agency (Foght et al., 2001).

*Fomitopsis* sp. IMER2 can treat black liquor through biological acidification, causing alkali lignin precipitation. Despite alkali lignin inhibiting fungal growth and acid production, it enhanced glucose consumption, suggesting a unique stress response. FTIR spectroscopy showed improvements in several functional groups of the resulting alkali lignin, aiding the fungus in utilizing available resources more effectively (Xiong et al., 2007). In the potato dextrose broth (PDB) medium, *F. pinicola* demonstrated a significant capacity for DDT degradation via different mechanisms (Purnomo et al., 2008, 2010). The addition of *P. aeruginosa* significantly enhanced the biodegradation of DDT by *F. pinicola*, achieving approximately 68% degradation after 7 days in Potato Dextrose Broth, compared to 42% by *F. pinicola* alone. The metabolites detected from this biodegradation included DDD, DDE, and DDMU (Sariwati and Purnomo, 2018). An intriguing discovery was found that a synergistic interaction between *F. pinicola* and the bacterium *Ralstonia pickettii* in degrading DDT, a highly toxic and recalcitrant pesticide that has been employed for eradicating malaria-carrying mosquitoes over an extended period (Purnomo et al., 2020). The co-culture of *F. pinicola* and *B. subtilis* for DDT degradation was explored, revealing that the addition of *B. subtilis* significantly enhanced the biodegradation of DDT by F. pinicola, resulting in the formation of DDE, DDD, and DDMU through distinct pathways (Sariwati et al., 2017). Research focusing on the bioremediation of wood contaminated with heavy metals highlighted the remarkable tolerance of *F palustris* to arsenic, copper, and chromium (Kartal et al., 2004; Hattori et al., 2015).

### 8.2 Wastewater treatment

**Figure 6:**
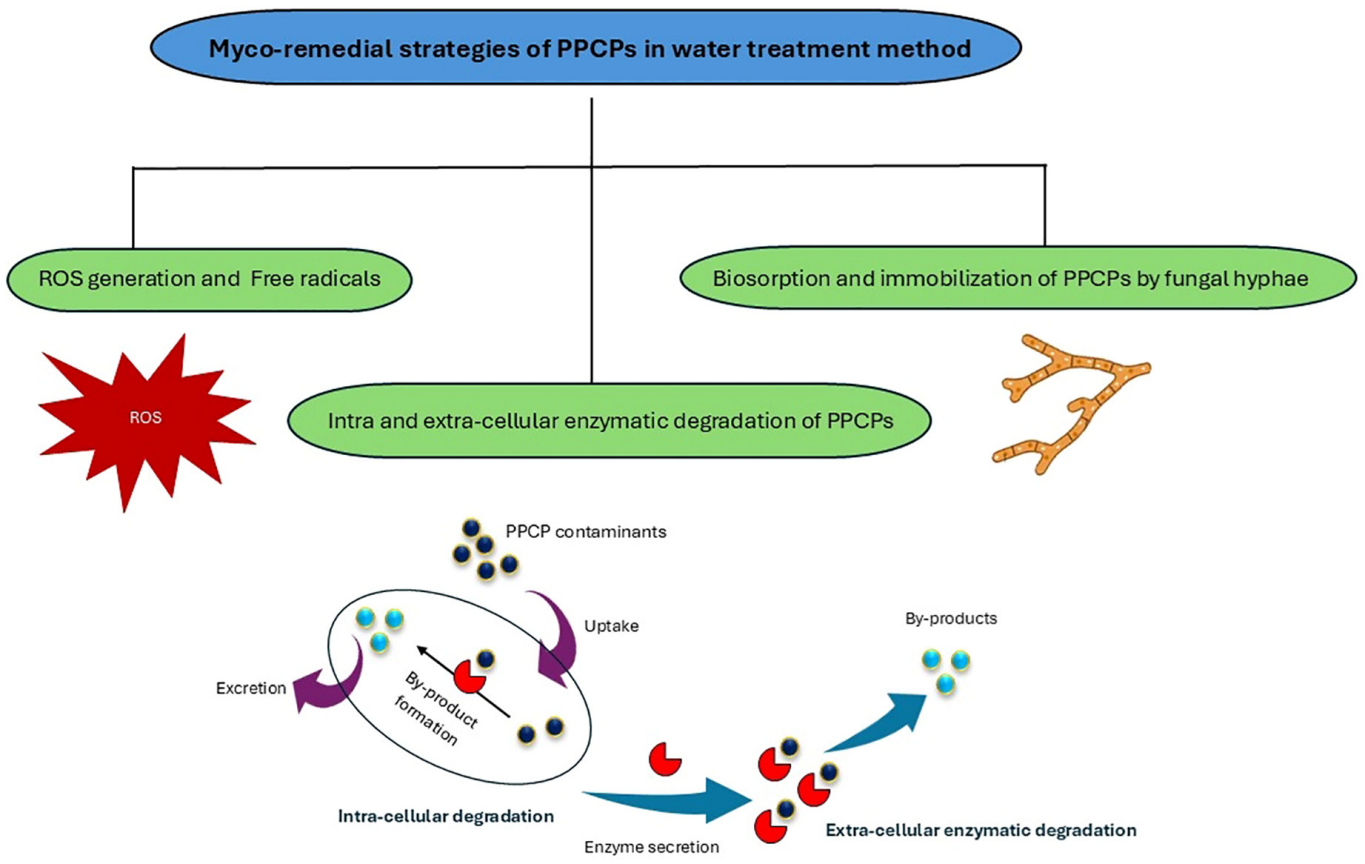
*Fomitopsis*-based remediation in waste-water treatment (Malik et al., 2023).

Species of *Fomitopsis* have been shown to have potential environmental applications, particularly in wastewater treatment. Their enzymatic systems and bioactive compounds contribute to the degradation of pollutants and the bioremediation of contaminated water sources (**Figure 6**). Exopolysaccharides (EPS) isolated from *F. castaneus* were assessed for their fermentation characteristics in simulated human intestinal environments. The EPS increased short-chain fatty acid (SCFA) production in both adult and child fecal extracts under anaerobic conditions. Furthermore, the addition of specific lactic acid bacteria enhanced SCFA content, with differing effects observed between adult and child models. *Fomitopsis palustris* hosts a unique, thermostable endoglucanase, which displayed remarkable efficiency on β-1, 4-glucans and has promising industrial applications (Cha et al., 2018). *Fomitopsis palustris*, played a key role in the transformation, where isobutylene, a valuable compound often derived from petroleum processes, was produced using a biomass source, isobutanol, via a novel dehydration process. The maximum isobutylene production rate observed was approximately 5.9 times higher than in previous studies, highlighting the efficiency of this method (Kim et al., 2019).

*Fomitopsis pinicola* exhibited a high decolorization rate and metabolic product analysis proposed three transformation pathways—demethylation, desulfonylation, and hydroxylation (Purnomo et al., 2019). Solid-state fermentation by *F. pinicola* improved the bioactivity and baking performance of wheat bran. Hence, *F. pinicola* was an excellent potential starter culture for wheat bran fermentation to develop functional whole grain foods (Tu et al., 2020). *Fomitopsis pinicola* has the potential to serve as a cost-effective adsorbent for treating low-concentration Cr (VI) wastewater (Pertile and Zamarsky, 2020). A novel laccase from *F. pinicola* was isolated and the enzyme exhibited the highest specific activity with ABTS. It effectively degraded various recalcitrant αs at different time intervals, suggesting its industrial potential (Park and Park, 2014). *Fomitopsis feei* effectively decolorized triphenylmethane dyes, especially basic fuchsin (98%). Lignolytic activity didn’t correlate with dye decolorization, however, Triphenylmethane reductase was the key enzyme. This eco-friendly method shows promise for treating dye industry effluents (Nidadavolu et al., 2013). *Fomitopsis rosea* demonstrated a higher effectiveness in decolorizing methylene blue (Jayasinghe et al., 2008). The use of dead *F. carnea* has proven effective in the biosorption of three cationic dyes—Orlamar Red BG (ORBG), Orlamar Blue G (OBG), and Orlamar Red GTL (ORGTL)—with high saturation capacities of 503.1, 545.2, and 643.9 mg/g, respectively. This process represents an effective method for wastewater treatment, as it removes harmful pollutants from contaminated water (Mittal and Gupta, 1996).

### 8.3 Industrial waste treatment

Understanding the mechanisms underlying brown rot decay opens up exciting prospects for applying these fungi in biotechnology. The enzymatic and non-enzymatic systems breaking down lignocellulose offer promising avenues for biomass bioprocessing, targeting the production of fuels and chemicals (Ray et al., 2010; Giles and Parrow, 2011; Arantes et al., 2012). The adsorption of methylene blue by *F. pinicola* from Kızılcahamam Işık Mountain, Turkey, was studied. Factors like dye concentration, adsorbent dose, pH, and temperature were tested. The adsorption followed the Langmuir isotherm and fit a pseudo-second-order kinetic model. Thermodynamic parameters were calculated, and the results suggest that *F. pinicola* can serve as a low-cost biosorbent for wastewater treatment (Sınağ et al., 2011). *Fomitopsis betulina* was evaluated for its ability to bioleach heavy metals, including Cu, Cr, and As, from wood preservatives, facilitated by the accumulation of metal-complexing oxalic acid (Sierra, 2007), and has a potential for As bioremediation in contaminated soils (Button et al., 2020). In addition, research has derived into the production of biomass-degrading enzymes such as cellulases, hemicellulases, and amylases (Krupodorova et al., 2014; Valášková and Baldrian, 2006a, 2006b). *Fomitopsis pinicola* effectively degrades polyvinyl alcohol (PVA) in quartz sand but not in liquid culture. Gel permeation chromatography revealed a decrease in the high-molecular-weight PVA fraction, with increased coloration due to low-molecular-weight PVA. Spectral analysis indicated a Fenton reaction-based degradation, suggesting the potential of F. pinicola for PVA degradation in woody wastes (Tsujiyama and Okada, 2013). *Fomitopsis pinicola*, a high-efficiency BGL-producing strain, was isolated and improved using thiamine (20 mg/L) supplementation. This led to a 3.7-fold increase in BGL activity (114.4 μmol/min/mg protein). The BGL-specific activity is remarkable, making *F. pinicola* valuable for industrial use, particularly in bio-energy production. The purified BGL exhibited exceptional catalytic efficiency, enhancing glucose production through biological processes (Joo et al., 2009).

### 8.4 Other biotechnological applications

#### 8.4.1 Fomitopsis palustris

*Fomitopsis palustris* degrades crystalline cellulose (Avicel) and produces cellulases capable of converting it into soluble sugars, with a cellulose conversion degree of 3.2% (Yoon and Kim, 2005). *Fomitopsis palustris* effectively performs sugar conversion, achieving a 40.6% yield within 72 hours when using rice straw as a substrate (Kim Y. et al., 2010). *Fomitopsis palustris* secretes oxalic acid during wood decay. Oxalate transport is ATP-dependent and inhibited by various substances. A cDNA, FpOAR, was isolated, and it appears to function as an oxalate transporter. FpOAR-transformants are resistant to oxalic acid, and FpOAR plays a crucial role in oxalate secretion during wood decay (Watanabe et al., 2010). *Fomitopsis palustris* was optimized for cellulase production from 11 wood rotting fungi. The optimized FPase activity reached 130.45 FPU/mL in an 8-day culture with 4.46 g/L of urea and 27.83 μL/L of Tween 80. The crude cellulase saccharified liquid hot water (LHW)-pretreated *Populus tomentosa*, released 25.15% reducing sugars after 72 hours, significantly higher than the 14.66% from untreated wood. This demonstrates the potential of *F. palustris* for cellulase production in woody biomass hydrolysis (Wang et al., 2012). The cost of bioethanol production from lignocellulosic materials is high due to delignification and saccharification processes. Consolidated bioprocessing (CBP) combines these with fermentation in one reactor, potentially reducing costs. The white rot fungus *Schizophyllum commune* was identified as a strong fermenter. When combined with *Bjerkandera adusta* and *F. palustris*, *B. adusta* enhanced ethanol production from cedar wood, while *F. palustris* released glucose but did not increase ethanol yield. The results suggest that using multiple fungi in CBP can effectively produce bioethanol from cellulosic materials (Horisawa et al., 2019). Two starch-degrading enzymes, FpAmy13A (α-amylase) and FpGLA15A (glucoamylase), were purified and characterized from *F. palustris*, and their corresponding genes were cloned. Variations in enzyme characteristics suggest distinct roles in wood starch degradation (Tanaka et al., 2020). *Fomitopsis palustris* CQ2018 effectively mobilized soil phosphorus, enhancing phosphorus availability in various soil types and promoting root growth in pepper (*Capsicum annuum*) plants. The fungal inoculation increased phosphorus uptake and fruit yield, improving fruit quality by raising levels of potassium and vitamin C while reducing nitrate content. This fungus shows potential as an environmentally friendly biofertilizer, with further research needed on its applications in different plants and soils (Peng and Huang, 2022).

#### 8.4.2 Fomitopsis pinicola

**Figure 7:**
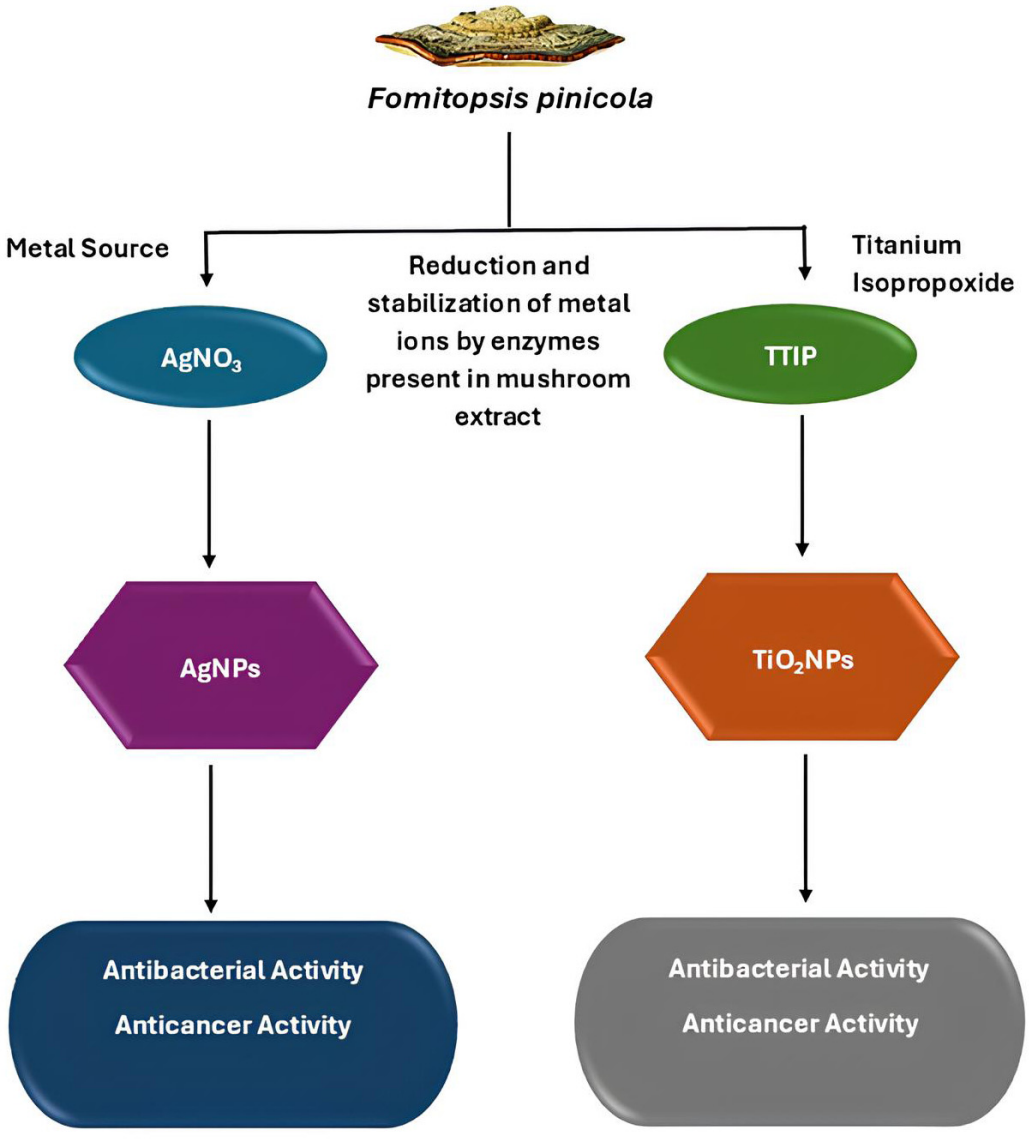
Benefits of *Fomitopsis-*based nanoparticles (Rehman et al., 2020).

Incorporating *F. pinicola* mycelium (CM) in bread dough fermentation improved its bioactive properties and bread quality. Concentrations of 30–40% CM maintained quality, reduced baking loss, and enhanced sensory characteristics, while 50% CM reduced loaf volume and crust brightness. In addition, CM delayed retrogradation and reduced mold growth during storage, with 30–40% CM identified as optimal for maintaining quality and offering potential anti-diabetic benefits (Seung-Hee, 2005). Cellulase from *F. pinicola* KMJ812, known for its high β-glucosidase activity, was immobilized on various resins, with Duolite A568 yielding the best results: 61.7% cellulase activity and 64.4% β-glucosidase activity. The optimal reaction conditions were 55°C and pH 4.0-4.5, slightly more stable than the unimmobilized enzyme. Notably, the immobilization improved the enzyme’s thermal stability, making it more suitable for industrial applications. The immobilized enzyme retained 98% of its activity after 72 hours at 50°C and 50% after eight uses at the same temperature (Shin et al., 2010b). *Fomitopsis pinicola* contains 30.11% chitin and yields 71.75% chitosan. The chitin is 72.5% acetylated, with a DTGmax of 341°C. Chitosan has a 73.1% deacetylation rate and a DTGmax of 265°C. Chitin exhibits a CrI of 52%, while chitosan has a CrI of 41%. Both show nano-fiber surface structures. *Fomitopsis pinicola* could serve as an alternative chitin and chitosan source due to its abundance (Kaya et al., 2015). Endoglucanase, with an apparent molecular weight of 32 kDa, was purified from *F. pinicola*). The bio-directed synthesis of titanium oxide and silver nanoparticles using *F. pinicola* was confirmed through various analytical techniques. These nanoparticles exhibited significant antibacterial and anticancer activities, with AgNPs demonstrating enhanced effects, highlighting their potential for biomedical applications (Rehman et al., 2020, **Figure 7**). The methanolic extract of *F. pinicola* was evaluated as a green corrosion inhibitor for 1018 carbon steel in 0.5 M sulfuric acid. The extract demonstrated 85% inhibition efficiency at 400 ppm, with thermodynamic analysis showing physical adsorption following the Langmuir isotherm. Solid-state fermentation of wheat bran by *F. pinicola* significantly improved its nutritional and sensory properties, increasing phenol, alkylresorcinol, and antioxidant content. The fermentation also enhanced the flavor, reduced phytic acid, and improved the texture of dough and bread, making it a promising method for enhancing wheat bran as a nutritious food ingredient (Tu et al., 2020). The extract formed a protective film on the steel surface, increasing polarization resistance at higher extract concentrations. GC-MS identified Dehydrergosterol and Phthalic acid as the main inhibitory compounds, supported by theoretical EHOMO and ELUMO values (Martinez-Gonzalez et al., 2024).

Brown rot fungi (*G. sepiarium*, *F. pinicola*, and *L. sulphureus*) filtrate saccharified biomass. *Fomitopsis pinicola* had high cellobiohydrolase activity, and combining *L. sulphureus* and *F. pinicola* enzymes increased sugar yield (Lee J. et al., 2008). Exoglucanase production from *Fomitopsis* sp. RCK2010 was optimized, resulting in a significant increase. The crude cellulase was then applied to saccharify pretreated *Prosopis juliflora*, releasing reducing sugars for bioethanol production (Deswal et al., 2012). **Table 7** outlines the biotechnological applications of various *Fomitopsis* species, focusing on their industrial, pharmaceutical, and environmental uses.

**Table 7:** Biotechnological applications of various species of *Fomitopsis*.

*Fomitopsis* Species	Study Focus	Applications/Bioactivity	Details/Outcomes	References
*Fomitopsis betulina*	Bioleaching of heavy metals	Bioleaching/Environmental Remediation	Effectively removed Cu, Cr, and As from wood preservatives, useful for bioremediation	Sierra (2007); Button et al. (2020)
*F. pinicola*	Adsorption of Methylene Blue	Wastewater treatment	Adsorption followed Langmuir isotherm; good biosorbent for methylene blue removal	Sınağ et al. (2011)
	Polyvinyl alcohol (PVA) degradation	Degradation of synthetic materials	Degraded PVA in quartz sand via Fenton reaction-based mechanism	Tsujiyama and Okada (2013)
	BGL activity enhancement	Bio-energy production	Thiamine increased BGL activity 3.7-fold, valuable for glucose production	Joo et al. (2009)
	Nutritional enhancement of wheat bran	Food industry	Fermentation increased phenolic content, antioxidant activity, and improved flavor	Tu et al. (2020)
	Endoglucanase purification	Enzyme purification/Industrial enzyme use	Purified endoglucanase had high specific activity, suitable for biomass degradation	Yoon et al. (2008b)
	Saccharification of biomass	Biomass conversion/Enzyme production	Increased sugar yield in biomass saccharification with high cellobiohydrolase activity	Lee J. et al. (2008)
	Bread dough fermentation with mycelium	Food industry/Improved bread quality	Enhanced bread quality and delayed mold growth at 30-40% concentration	Seung-Hee (2005)
	Corrosion inhibition	Green corrosion inhibition	Methanolic extract showed 85% inhibition efficiency for carbon steel	Martinez-Gonzalez et al. (2024)
	Immobilization of cellulase for industrial use	Cellulase immobilization/Industrial enzyme stability	Immobilized cellulase had high thermal stability and retained activity after repeated use	Shin et al. (2010b)
*F. palustris*	Purification of starch-degrading enzymes	Starch degradation/Industrial enzyme production	Purified α-amylase and glucoamylase enzymes, specialized roles in wood starch degradation	Tanaka et al. (2020)
	Bioethanol production	Bioethanol production/Consolidated bioprocessing (CBP)	Released glucose but didn’t improve ethanol yield from cellulosic materials	Horisawa et al. (2019)
	Phosphorus mobilization for plant growth	Biofertilizer	Increased phosphorus uptake, root growth, and fruiting yield in pepper plants	Peng and Huang (2022)
	Cellulase production and sugar conversion	Biomass degradation	Converted crystalline cellulose into soluble sugars, achieving a 40.6% yield	Kim Y. et al. (2010); Yoon and Kim (2005)
	Oxalate secretion during wood decay	Wood decay/Enzyme secretion	Secretes oxalic acid during decay, regulated by various substances	Watanabe et al. (2010)
	Cellulase production optimization	Biomass hydrolysis/Cellulase production	Optimized cellulase production, effectively hydrolyzed pretreated *Populus tomentosa*	Wang et al. (2012)
*Fomitopsis* sp. RCK2010	Exoglucanase production optimization	Exoglucanase production/Saccharification	Optimized exoglucanase, saccharified pretreated *Prosopis juliflora*, releasing sugars for bioethanol	Deswal et al. (2012)

## 9 Conclusions

Species of *Fomitopsis*, with the diverse bioactive compounds they produce, hold significant promise in traditional and modern medicinal applications. This comprehensive review has highlighted the potent antioxidant, immunomodulatory, and anti-inflammatory properties of key compounds such as polysaccharides, triterpenoids, and phenolic substances found within *Fomitopsis*. These properties underscore the therapeutic potential of *Fomitopsis* in cancer treatment and immune system support and emphasize its role as a valuable source of natural antioxidant supplements. However, there is no documented evidence of significant toxicity in *Fomitopsis* species based on available literature. Comprehensive studies on their long-term safety and potential adverse effects are limited. Hence, more research is needed to fully understand their toxicity profile and ensure safe therapeutic use.

Moreover, the exploration of biotechnological approaches, including advanced cultivation, extraction, and purification techniques, is a fascinating area of study that reveals the growing interest in optimizing the production and utilization of *Fomitopsis* bioactive compounds. The challenges and prospects in this field underscore the importance of continued research and innovation, inviting the scientific community to contribute to unlocking the full potential of *Fomitopsis* in medicine and biotechnology. In conclusion, *Fomitopsis* stands out as a genus with substantial health benefits and biotechnological value. Future research should focus on further elucidating the molecular mechanisms of its bioactive compounds, enhancing biotechnological methods, and expanding its applications in various therapeutic areas, paving the way for new, effective treatments and health-promoting products.
